# Itraconazole for COVID-19: preclinical studies and a proof-of-concept randomized clinical trial

**DOI:** 10.1016/j.ebiom.2021.103288

**Published:** 2021-03-19

**Authors:** Laurens Liesenborghs, Isabel Spriet, Dirk Jochmans, Ann Belmans, Iwein Gyselinck, Laure-Anne Teuwen, Sebastiaan ter Horst, Erwin Dreesen, Tatjana Geukens, Matthias M. Engelen, Ewout Landeloos, Vincent Geldhof, Helga Ceunen, Barbara Debaveye, Bert Vandenberk, Lorenz Van der Linden, Sofie Jacobs, Lana Langendries, Robbert Boudewijns, Thuc Nguyen Dan Do, Winston Chiu, Xinyu Wang, Xin Zhang, Birgit Weynand, Thomas Vanassche, Timothy Devos, Geert Meyfroidt, Wim Janssens, Robin Vos, Pieter Vermeersch, Joost Wauters, Geert Verbeke, Paul De Munter, Suzanne J.F. Kaptein, Joana Rocha-Pereira, Leen Delang, Eric Van Wijngaerden, Johan Neyts, Peter Verhamme

**Affiliations:** a Laboratory of Virology and Chemotherapy, Department of Microbiology, Immunology and Transplantation, Rega Institute for Medical Research, KU Leuven, Leuven, Belgium; b The Outbreak Research Team, Department of Clinical Sciences, Institute of Tropical Medicine, Antwerp, Belgium; c Pharmacy Department University Hospitals Leuven and Department of Pharmaceutical and Pharmacological Sciences, KU Leuven, Belgium; d KU Leuven – University of Leuven & Universiteit Hasselt, I-BioStat, Leuven, Belgium; e Department of Respiratory Diseases, UZ Leuven and CHROMETA, Research group BREATHE, KU Leuven, Leuven, Belgium; f Laboratory of Angiogenesis and Vascular Metabolism, Center for Cancer Biology, VIB, Department of Oncology, Leuven Cancer Institute, KU Leuven, Leuven, Belgium; g Clinical Pharmacology and Pharmacotherapy Unit, Department of Pharmaceutical and Pharmacological Sciences, KU Leuven, Belgium; h Department of Oncology, Laboratory for Translational Breast Cancer Research, KU Leuven, Belgium; i Department of Cardiovascular Sciences, UZ and KU Leuven, Belgium; j Department of Oncology, Laboratory for molecular Cancer biology, VIB-KU Leuven, Belgium; k Department of General Internal Medicine, UZ Leuven and Department of Microbiology, Immunology and Transplantation, KU Leuven, Belgium; l Translational Cell and Tissue Research, Department of Imaging and Pathology, KU Leuven, Belgium; m Department of Hematology, UZ Leuven and Department of Microbiology, Immunology and Transplantation, Laboratory of Molecular Immunology (Rega Institute), KU Leuven, Belgium; n Department and Laboratory of Intensive Care Medicine, UZ and KU Leuven, Belgium; o Department of Cardiovascular Sciences and Clinical Department of Laboratory Medicine, KU Leuven, Belgium; p Medical Intensive Care Unit, UZ Leuven and Department of Microbiology, Immunology and Transplantation, KU Leuven, Belgium

**Keywords:** COVID-19, SARS-CoV-2, itraconazole, drug repurposing, antivirals

## Abstract

**Background:**

The antifungal drug itraconazole exerts *in vitro* activity against SARS-CoV-2 in Vero and human Caco-2 cells. Preclinical and clinical studies are required to investigate if itraconazole is effective for the treatment and/or prevention of COVID-19.

**Methods:**

Due to the initial absence of preclinical models, the effect of itraconazole was explored in a clinical, proof-of-concept, open-label, single-center study, in which hospitalized COVID-19 patients were randomly assigned to standard of care with or without itraconazole. Primary outcome was the cumulative score of the clinical status until day 15 based on the 7-point ordinal scale of the World Health Organization. In parallel, itraconazole was evaluated in a newly established hamster model of acute SARS-CoV-2 infection and transmission, as soon as the model was validated.

**Findings:**

In the hamster acute infection model, itraconazole did not reduce viral load in lungs, stools or ileum, despite adequate plasma and lung drug concentrations. In the transmission model, itraconazole failed to prevent viral transmission. The clinical trial was prematurely discontinued after evaluation of the preclinical studies and because an interim analysis showed no signal for a more favorable outcome with itraconazole: mean cumulative score of the clinical status 49 *vs* 47, ratio of geometric means 1.01 (95% CI 0.85 to 1.19) for itraconazole *vs* standard of care.

**Interpretation:**

Despite *in vitro* activity, itraconazole was not effective in a preclinical COVID-19 hamster model. This prompted the premature termination of the proof-of-concept clinical study.

Funding: KU Leuven, Research Foundation - Flanders (FWO), Horizon 2020, Bill and Melinda Gates Foundation

**Research in Context**

**Evidence before this study**

Before initiation of this study trial, in March, 2020, we searched PubMed, ClinicalTrials.gov, EudraCT, bioRxiv and medRxiv using the terms “itraconazole” and “SARS-CoV-2”. However, this search did dot not retrieve any results. When searching for “itraconazole” AND “antiviral activity” we found several studies showing significant in vitro activity of itraconazole against multiple RNA viruses, including feline coronavirus and influenza virus. In a lethal influenza mouse model, administration of itraconazole improved survival.

**Added value of this study**

After discovery of its antiviral activity against SARS-CoV-2, itraconazole was evaluated in a proof-of-concept clinical study in hospitalized patients with COVID-19 and in a preclinical hamster model of acute infection and transmission. Itraconazole was unable to reduce SARS-CoV-2 viral load in infected hamsters or improve clinical outcome in COVID-19 patients.

**Implications of all the available evidence**

Itraconazole should not be used in the treatment of COVID-19. Now that preclinical COVID-19 models are available, antiviral drug candidates should undergo preclinical testing before use in clinical trial or clinical practice.

Alt-text: Unlabelled box

## 1 Introduction

When the COVID-19 pandemic hit Europe in February 2020, no treatment against SARS-CoV-2 was available. At our institute, several drug libraries were screened with a high-throughput screening test for *in vitro* activity against the SARS-CoV-2 virus, with the goal to repurpose drugs. This drug screening revealed antiviral activity of itraconazole and its metabolite hydro-itraconazole [1], comparable with the *in vitro* antiviral activity of hydroxychloroquine. This activity was also confirmed by others [2] (both reports are at the time of writing available as preprints only).

Itraconazole was previously demonstrated to be active against several viruses, including the feline coronavirus [3] and influenza A virus [4]. Itraconazole even increased survival in an influenza mouse model [4]. Itraconazole has a well-known safety profile and generic preparations are available, making it an attractive candidate for drug repurposing. In addition, itraconazole accumulates well in lung tissue, although its low oral bioavailability is of concern [5].

At the onset of the pandemic, preclinical animal infection models to investigate the efficacy of antiviral drugs were not available. Given the potential antiviral activity of itraconazole, we therefore launched a pilot clinical trial to test its efficacy and safety in hospitalized patients with moderate to severe COVID-19. In parallel, we developed a hamster SARS-CoV-2 infection model to explore the potential antiviral effect of itraconazole and other agents [6].

Here, we report on the hamster studies with itraconazole both in an acute infection and viral transmission model. Next, the results of the pilot proof-of-concept trial in hospitalized COVID-19 patients are discussed.

## 2 Methods

### 2.1 In vitro antiviral assay

The SARS-CoV-2 strain used in this study was BetaCov/Belgium/GHB-03021/2020 (EPI ISL 407976|2020-02-03), which was isolated from a Belgian patient returning from Wuhan in February 2020 [7]. The SARS-CoV-2 antiviral assay is derived from the previously established SARS-CoV assay [8]. In this assay, fluorescence of VeroE6-eGFP cell cultures declines after infection with SARS-CoV-2 due to a cytopathogenic effect. In the presence of an antiviral compound, the cytopathogenicity is inhibited and the fluorescent signal maintained. The compounds were added in serial dilutions to the cells one day before infection with SARS-CoV-2. Five days after infection eGFP fluorescence was assessed with high content imaging. Additional details can be found in the Supplementary Methods.

### 2.2 Preclinical studies

The institutional Ethical Committee approved all animal experiments (license P065-2020). Both the acute infection hamster model and the transmission model have been described in detail elsewhere [6,9].

**Figure 1:**
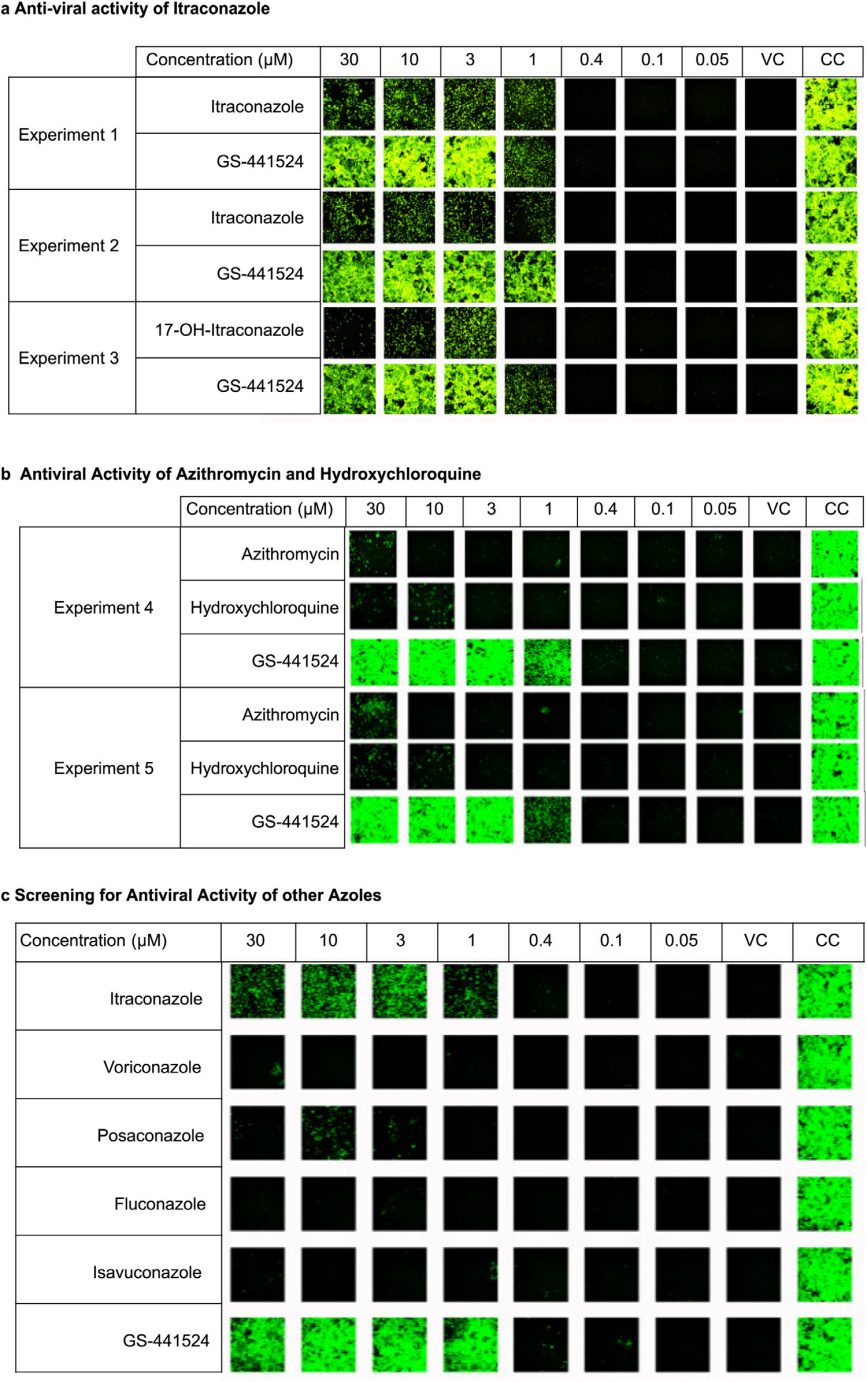
**In vitro antiviral activity. (a)** Antiviral activity of itraconazole and hydroxy-itraconazole at different concentrations in the SARS-CoV-2 / VeroE6-eGFP assay system. Green fluorescence indicates cells surviving SARS-CoV-2 infection. Itraconazole was tested in two independent experiments while hydroxy-itraconazole (17-OH-itraconazole) was tested in one experiment. **(b)** Antiviral activity of azithromycine, hydroxychloroquine and GS-441524 in two independent experiments. **(c)** Antiviral activity of itraconazole and other azoles different concentrations in the SARS-CoV-2 / VeroE6-eGFP assay system. VC indicates “Virus Control” i.e. infected untreated cultures; CC indicates “Cell Control” i.e. uninfected untreated cultures; GS-441524 is the parent nucleoside of remdesivir.

In the acute infection model (Figure 1a), female Syrian gold hamsters, six to ten weeks of age, were inoculated intranasally with 2×10^6^ TCID_50_ of SARS-CoV-2 in a high-containment A3 facility. This inoculum was chosen as it consistently induced high viral replication and lung pathology in previous experiments [6,9]. Hamsters were randomly assigned to treatment with itraconazole or vehicle (equal volume). Treatment was initiated one hour before infection and administered twice daily by gavage (10 mg/mL) at a dosage of 30 mg/kg/day (n=2×4) or 70 mg/kg/day (n=2×6). Hamsters were daily monitored for behavior and weight. Four days after the virus instillation, the animals were euthanized and lungs, stool, ileum, and plasma samples were analyzed. As primary outcome viral load in these tissues was quantified by RT-qPCR and end-point virus titration. A histological scoring system graded severity of pulmonary infection. Itraconazole concentrations in hamsters were measured at sacrifice (plasma and lung), which was kindly performed by Johnson & Johnson as described previously [10].

In the SARS-CoV-2 transmission hamster model (Figure 1h), index hamsters were first infected with SARS-CoV-2 (2×10^6^ TCID_50_) (n=2×5). The next day these index hamsters were cohoused with uninfected sentinel hamsters (n=2×5). Sentinel hamsters were started on itraconazole (70 mg/kg/day) or vehicle one day prior to cohousing. Infected index hamsters were sacrificed four days post infection, and sentinel hamsters four days after co-housing. One index hamster that received itraconazole, was euthanized prematurely due to complications with the gavage. The number of animals used per study (4-6) was determined by power calculations based on our previous experience with this hamster model [6,9], and took in account housing capacity. All experimental animals were of the same age and gender as their controls and were housed in the same conditions to minimize confounders. Researchers were not blinded to treatment conditions, except for individuals performing qPCR and viral titration.

### 2.3 Clinical study

#### 2.1.1 Study design

The Direct Antivirals Working against new Corona virus (DAWn)-Itraconazole study was an open-label, randomized, single-center pilot trial in hospitalized COVID-19 patients. The trial was conducted at the University Hospitals Leuven, Belgium, between March and June 2020. The study compared standard of care with or without itraconazole in a 1:1 randomization. Standard of care was based on guidelines of national and international organizations and outlined in an institutional policy.

The study was conducted in accordance with the International Conference on Harmonization Guidelines for Good Clinical Practice and the Declaration of Helsinki. The protocol was approved by the institutional Ethics Committee and by the Belgian Federal Agency for Medicines and Health Products (EudraCT 2020-001243-15). The trial was part of the DAWn clinical studies [11], [12], [13]. An independent Data Safety Monitoring Board (DSMB) reviewed trial safety outcomes. The full protocol and statistical analysis plan are available in Supplement.

#### 2.1.2 Patients

Hospitalized patients aged 18 years or older with COVID-19, confirmed by PCR or typical chest CT-scan, were eligible if they displayed at least one of the following features: radiographic infiltrates, Sp02 ≤ 94% on room air, or requiring supplemental oxygen. Subjects were excluded in case of elevated liver tests (ALT/AST > 5 times the upper limit of normal), pregnancy or breast feeding, heart failure with severely reduced ejection fraction (≤ 30%), or concomitant treatment with lopinavir/ritonavir or potent CYP450 inducers.

Because of safety concerns due to the risk of transmission of SARS-CoV-2, verbal informed consent in the presence of an independent witness was obtained during hospitalization of all patients. Written informed consent was obtained after discharge from quarantine.

#### 2.1.3 Interventions

Subjects were assigned to standard of care with or without itraconazole. Itraconazole was administered as capsules or as an oral solution with a loading dose of 200 mg three times per day for the first 3 days, followed by 200 mg twice daily. Itraconazole was continued for at least 10 days and up to 14 days if patients remained hospitalized. Itraconazole capsules were administered with a meal, whereas solution was given at least 2 hours before or 1 hour after a meal. Concomitant treatment with proton-pump inhibitors was avoided in patients treated with capsules.

Standard of care was defined by the Belgian COVID-19 guidelines [14], which at the time of recruitment included supportive treatment, broad spectrum antibiotics (ceftriaxone) and hydroxychloroquine .

Outcomes were assessed for 28 days. Clinical parameters and laboratory tests that where part of routine clinical care were captured from the electronic patient files. If feasible a follow-up nasopharyngeal swab was obtained on day 6. When discharged, patients were contacted by phone on day 15 and day 29 to verify their clinical status.

#### 2.1.4 Randomization and masking

Randomization (1:1) with a computerized system was stratified according to disease severity. The study was open label without blinding for patients, healthcare workers or investigators. Throughout the study, the trial statisticians were blinded to the different treatments. They were not given direct access to the database and only received data from which any information regarding treatment allocation was removed (e.g. all treatment data, plasma concentrations…). The randomization schedule was kept on a separate location, inaccessible to the statisticians, and was only sent at the time of database lock.

#### 2.1.5 Outcomes

The primary outcome was defined as cumulative clinical status on day 15. This endpoint consists of the sum of daily clinical status scores on the 7-point WHO ordinal scale from day 1 to 15 included. The WHO ordinal scale consists of the following 7 categories: 1) not hospitalized, no limitations on activities; 2) not hospitalized, limitations on activities; 3) hospitalized, not requiring supplemental oxygen; 4) hospitalized, requiring supplemental oxygen; 5) hospitalized, on non-invasive ventilation or high flow oxygen devices; 6) hospitalized, requiring extracorporeal membrane oxygenation (ECMO) or invasive mechanical ventilation; and 7) death.

The secondary outcome was defined as time to sustained clinical improvement or live discharge, whichever comes first, whereby a sustained clinical improvement is defined as an improvement of more than 2 points on the 7-point ordinal scale versus the highest value of day 0 and 1 and sustained for at least 3 days. Other secondary outcomes included: time to events (admission to ICU, death, discharge); mortality on day 29, duration of supplemental oxygen, need for and duration of mechanical ventilation, duration of hospitalization, duration of intensive care stay, daily National Early Warning Score (NEWS). Safety outcomes included adverse events (AE) graded as grade 4 or 5 or serious adverse events (SAE) and ECG monitoring. Quantitative PCR for SARS-CoV-2 in (nasopharyngeal) swab on day 1 and day 6 were exploratory outcomes.

For pharmacokinetic evolution, itraconazole and its main metabolite, hydroxy-itraconazole, trough concentrations were measured at different time points with liquid chromatography tandem mass spectrometry (Waters Acquity TDQ system with Recipe ClinMass® antimycotics kit). The trough concentrations measured before and after 72 hours after initiation of treatment were defined as early and late exposure, respectively.

#### 2.1.6 Statistical analysis

Preclinical studies were analyzed with GraphPad Prism (GraphPad Software, Inc.); the non-parametric Mann Whitney U-test was used to ascertain statistical significance; a P value ≤0.05 was considered significant.

Analyses of the clinical data were performed with SAS software version 9.41. All treatment comparisons made using two-sided tests at a significance level of 5% and were adjusted for disease severity, to account for the stratified randomization. For all outcomes, the treatment effect was estimated using an appropriate measure (e.g. hazard ratio, treatment difference, etc.) and presented along with its 95% confidence interval. All analyses were performed on this Full Analysis Set (FAS) which included all randomized patients, with the exception of randomized patients that violated the following eligibility criteria:

a) No confirmed diagnosis of COVID-19.
b) Known drug-drug interaction with Intraconazole.

Missing Clinical Status data up to Day 29 were accounted for by means of multiple imputation, using a total of 100 imputations as described by Rubin [15]. Missing in-hospital clinical status scores were imputed using the fully conditional specification method [16] for a multinomial logistic regression, in a consecutive manner. i.e. first missing Day 1 scores were imputed based on Day 0 scores and clinical variables (randomized treatment, baseline disease severity, oxygen flow and CRP on previous day); then each consecutive day was imputed using scores of the 5 previous days and the same clinical variables. The cumulative clinical status was analyzed using a general linear model adjusted for the status at baseline and disease severity after log-transformation, thus yielding the ratio of geometric means between the treatment groups. Time-to-event data were analyzed using a Cox regression or, in the presence of competing risks, a Fine&Gray regression model [17], yielding hazard ratios and subdistribution hazard ratios, respectively.

Full details of statistical analyses are provided in the Statistical Analysis Plan, which is provided in the online Supplement.

Sample size estimation was based on the clinical scores on day 7 and day 15 from previously published COVID-19 clinical trial data [18]. The mean cumulative clinical severity score on day 15 was estimated to be 60, with a standard deviation of 20. With a power of 0.8 and an alpha of 0.05, sample size estimates to detect a 5-point difference and an 8-point difference in cumulative clinical severity score required 502 patients (251 in each group) and 196 patients (2 times 98), respectively. No formal interim analyses for efficacy or futility were foreseen. Descriptive analysis of the pharmacokinetic analysis was carried out in R Statistics (version 3.5.1, R Core Team).

#### 2.1.7 Role of the funding source

The funder had no role in the collection, analysis, and interpretation of data; in the writing of the report; and in the decision to submit the paper for publication. The corresponding author had full access to all the data in the study and had final responsibility for the decision to submit for publication. All authors vouch for the accuracy and completeness of the data.

## 3 Results

### 3.1 In vitro antiviral activity of itraconazole and other azoles

We confirmed the activity of itraconazole and hydroxy-itraconazole in an *in vitro* antiviral assay using SARS-CoV-2 infected VeroE6-eGFP cells (Figure 1a). Although the antiviral activity is less than that of GS-441524 (the parent nucleoside of remdesivir), it remains significant up to concentrations as low as ~1 µM for itraconazole and 3 µM for hydroxy-itraconazole and was comparable to that of hydroxychloroquine (Figure 1b). In search of the optimal drug repurposing candidate, we tested additional azoles with better bioavailability. However, we did not detect antiviral activity for posaconazole, voriconazole, isavuconazole and fluconazole against SARS-CoV-2 (Figure 1c)

Tentative target concentrations for the preclinical and clinical trial were based on the lowest active concentrations for itraconazole and hydroxy-itraconazole activity in the *in vitro* experiment (1 µM and 3 µM). Taking into account the molecular weight and previously published lung/plasma ratios, plasma concentrations of 0.5 to 1 mg/L and 0.75 to 1 mg/L were targeted for itraconazole and hydroxy-itraconazole, respectively [5,19.

### 3.2 Preclinical studies in the COVID-19 hamster model

**Figure 2:**
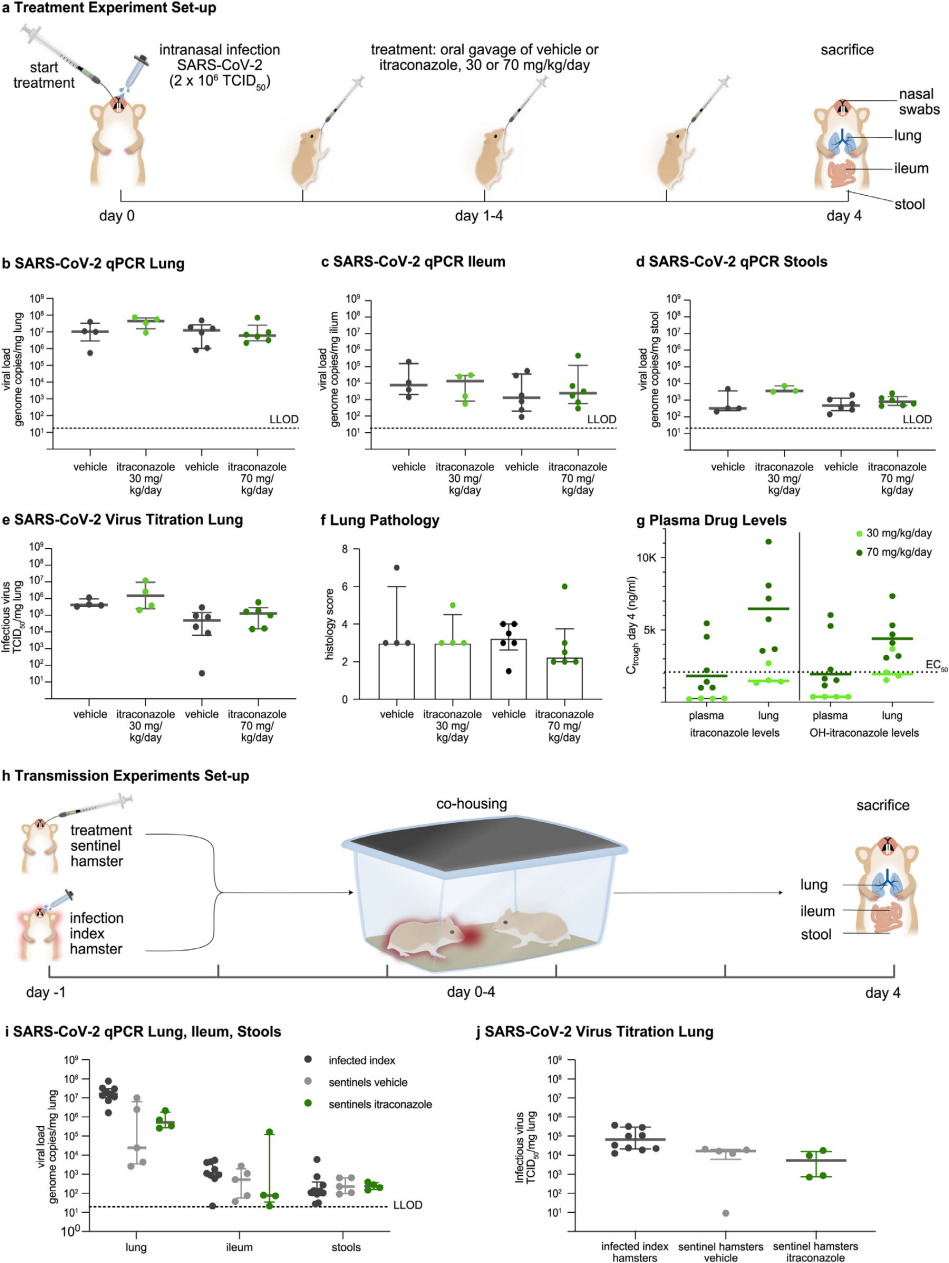
**Preclinical Evaluation of Itraconazole. (a)** Acute infection model set-up. Hamsters (n=18) were intranasally infected with SARS-CoV-2 (2x10^6^ 50% tissue culture infectious dose, (TCID_50_)). Treatment with vehicle or itraconazole 30 or 70 mg/kg/day in two gifts via oral gavage, started one hour before infection. Hamsters were sacrificed at day 4. **(b-d)** Viral RNA levels quantified by RT-qPCR in **(b)** lungs, **(c)** ileum and **(d)** stools. **(e)** Lung infectious viral load assessed by endpoint dilution on cell cultures and expressed as TCID_50_ per mg of lung tissue. **(f)** Pathology score of the severity of inflammation on H&E stained lung sections at day 4. **(g)** Day 4 plasma and lung trough concentrations (C_trough_) of itraconazole and hydroxy-itraconazole in hamsters treated with either 30 or 70 mg/kg/day itraconazole. The dashed line indicates the EC_50_ of itraconazole against SARS-CoV-2. **(h)** Design of the viral transmission study: From day -1 on sentinel hamsters (n=2x5) received itraconazole (70 mg/kg/day) or vehicle. Index hamsters (n=2x5) were intranasally infected with SARS-CoV-2 (2x10^6^ TCID_50_) on day -1. From day 0 onwards index and sentinel hamsters were co-housed, while treatment of sentinel hamsters continued (n=18). **(i)** Viral RNA levels quantified by RT-qPCR on day 4 in index hamsters and sentinel hamsters in lung, ileum and stool. **(j)** Lung infectious viral load in the lung expressed as TCID_50_ per mg of lung tissue. Bars represent median ± interquartile range (IQR). Data were analysed with the two-sided Mann-Whitney *U*-test. No statistically significant differences were found. LLOD denotes lower limit of detection.

In the acute COVID-19 hamster model (Figure 2a), the two doses of itraconazole (30 mg/kg/day and 70 mg/kg/day), administered by oral gavage twice-daily for four consecutive days, did not reduce viral RNA load in the lungs, ileum or stools (Figure 2b-d), nor infectious virus titers in the lungs (median Tissue Culture Infectious Dose, TCID_50_; Figure 2e). In addition, treatment with itraconazole did not mitigate pulmonary inflammation on histology (Figure 2f).

The tested dosing regimens were based on pharmacokinetic analyses of itraconazole in hamsters (data not shown). The dosage of 30 mg/kg/day was well tolerated, whereas hamsters treated with 70 mg/kg/day showed weight loss, pointing to toxicity (Supplementary Figure 1a). To ensure that the lack of efficacy was not due to suboptimal drug levels in infected hamsters, we determined trough concentrations of itraconazole and hydroxy-itraconazole in plasma and lung tissue at sacrifice (i.e. 12 hours after the last dose) (Figure 2g). For the high-dose regimen, lung concentrations were above the EC_50_ against SARS-CoV-2 (3 µM or 2115 ng/ml) and close to the EC_90_ level (defined as 3 times the EC_50_ level). Hence, despite sufficient exposure, no *in vivo* activity was observed. In addition, there was no relationship between lung itraconazole exposure and reduction in viral load (Supplementary Figure 1b).

Besides, treatment with itraconazole did not reduce viral shedding from the nose of infected hamsters (Supplementary Figure 2c). Likewise, in the SARS-CoV-2 transmission experiments (Figure 1h), prophylactic treatment with itraconazole did not protect sentinel hamsters from infection. SARS-CoV-2 viral loads in lungs, ileum and stools of untreated and treated sentinel animals did not differ (Figure 2i,j).

### 3.3 Patients

**Figure 3:**
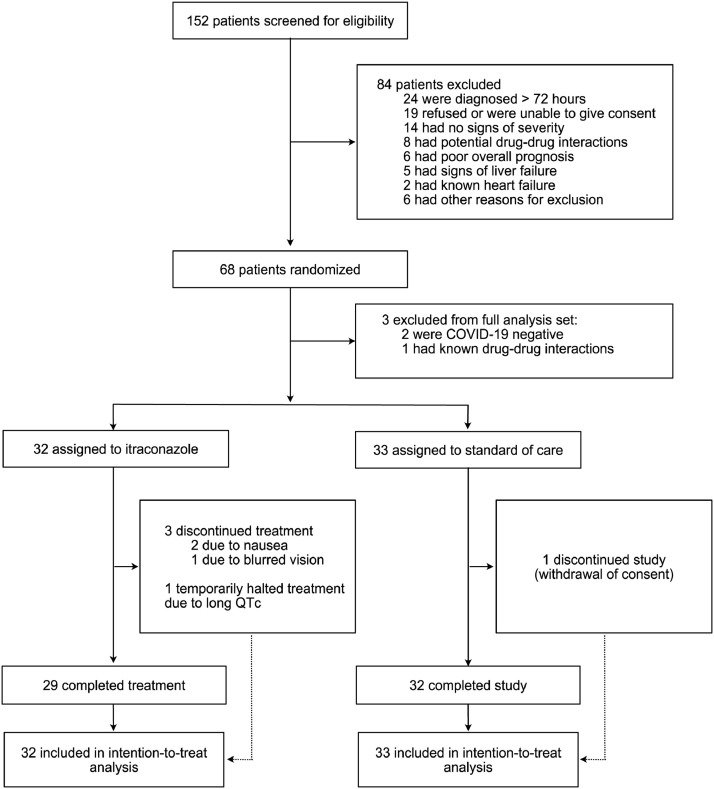
Enrolment and Randomisation of Clinical Trial Participants

Patients were recruited in March and April 2020. Of the 152 patients who were screened for eligibility, 68 were enrolled. Three patients were excluded from the Full Analysis Set: two patients had no documented COVID-19 and one patient did not fulfill in- and exclusion criteria because of drug-drug interactions (Figure 3). Eight patients were treated with capsules, 16 with oral solution and 9 with a combination of both. Three patients prematurely discontinued treatment with itraconazole: two patients reported nausea and one disturbed vision. One patient in the standard group prematurely discontinued the trial (withdrawal of consent)

The study was put on hold by the DSMB when 68 patients had been randomized in order to evaluate pharmacokinetic data. After reviewing the preliminary pharmacokinetic and the preclinical data, the Steering Committee decided not to restart the study.

There were no major differences in baseline characteristics (Table 1). The mean age of participants was 62 (SD=11) years of age and 63% were men. The majority of patients presented with dyspnea (80%), fever (72%) or cough (65%). The median time of onset of symptoms to inclusion in the trial was 8 days (IQR=5 to 10).

**Table 1:** Demographics and Baseline Characteristics of the Clinical Trial Participants

**Characteristic**	**Itraconazole (N=32)**	**Standard Care (N=33)**	**Total (N=65)**
Age (years) – mean (SD)	62 (10)	63 (13)	62 (11)
Female – no. (%)	11 (34%)	13 (39%)	24 (37%)
Body weight [kg]- mean (SD)	84 (16)	80 (11)	82 (14)
Comorbidities			
At least one comorbidity – no. (%)	22 (69%)	19 (59%)	41 (64%)
Diabetes – no. (%)	7 (22%)	8 (24%)	15 (23%)
Hypertension – no. (%)	16 (50%)	11 (33%)	27 (42%)
Heart failure – no. (%)	1 (3%)	2 (6%)	3 (5%)
History of cancer – no. (%)	2 (6%)	8 (25%)	10 (16%)
Chronic pulmonary disease – no. (%)	1 (3%)	1 (3%)	2 (3%)
Chronic liver disease – no. (%)	4 (13%)	2 (6%)	6 (9%)
Chronic kidney disease – no. (%)	3 (9%)	2 (6%)	5 (8%)
DNR code at admission			
DNR0 – no. (%)	30 (94%)	29 (88%)	59 (91%)
DNR1 – no. (%)	1 (3%)	0 (0%)	1 (2%)
DNR2 – no. (%)	1 (3%)	4 (12%)	5 (8%)
Symptoms			
Dyspnea – no. (%)	28 (88%)	24 (73%)	52 (8%)
Fever – no. (%)	22 (69%)	25 (76%)	47 (7%)
Cough – no. (%)	19 (5%)	23 (70%)	42 (65%)
Diarrhea or vomiting – no. (%)	18 (56%)	10 (30%)	28 (43%)
Days from symptom onset to randomisation – median (IQR)	8 (5 to 10)	8 (6 to 11)	8 (5 to 10)
Imaging			
Abnormal CT – no. (%)	32 (100%)	32 (97%)	64 (99%)
Bilateral pneumonia on CT – no. (%)	31 (97%)	31 (97%)	62 (97%)
Signs			
Oxygen saturation – mean (SD)	94.8 (3.0)	93.9 (6.6)	94.3 (5.1)
Respiratory rate – mean (SD)	21.5 (5.2)	21.4 (4.6)	21.4 (4.8)
Needing oxygen support at admission – no. (%)	24 (75%)	18 (55%)	42 (65%)
Systolic blood pressure – mean (SD)	131.9 (22.6)	137.0 (21.6)	134.5 (22.1)
Laboratory findings			
C-reactive protein [mg/L] – median (IQR)	70.0 (15.7 to 110.3)	88.0 (36.4 to 121.0)	73.7 (28.6 to 17.1)
White blood cell count [10^9^/L] – mean (SD)	6.5 (2.3)	6.5 (2.3)	6.5 (2.3)
Hemoglobin [g/dL] – mean (SD)	14.1 (2.0)	13.8 (2.0)	13.9 (2.0)
Platelet count [10^9^/L] – mean (SD)	234.2 (94.8)	236.4 (116.1)	235.3 (105.3)
Serum creatinine [mg/dL] – median (IQR)	1.0 (0.8 to 1.2)	0.8 (0.7 to 1.1)	0.9 (0.7 to 1.1)
AST [U/L] – median (IQR)	44.5 (27.0 to 63.0)	37.0 (29.0 to 70.0)	43.0 (27.0 to 68.0)
ALT [U/L] – median (IQR)	33.0 (22.0 to 54.0)	30.0 (19.0 to 54.0)	32.0 (20.5 to 54.0)
D-Dimer [ug/L] – median (IQR)	776.0 (513.0 to 1160.0)	979.0 (766.0 to 1199.0)	914.5 (563.0 to 1199.0)
Ferritin [ug/L] – median (IQR)	839.5 (323.0 to 1430.0)	846.5 (399.5 to 1144.0)	839.5 (395.0 to 1299.0)
ECG			
Arrhythmia on ECG – no. (%)	2 (7%)	5 (15%)	7 (11%)
QTc [ms] – mean (SD)	440.1 (29.2)	437.9 (25.1)	439.0 (27.0)

SD denotes standard deviation, DNR do not resuscitate, IQR interquartile range, CT chest tomography, ECG electrocardiogram, QTc corrected QT interval.

All participants required hospitalization, 89% required oxygen supplementation and 29% needed ICU admission. All but one patient received hydroxychloroquine, part of the Belgian standard of care at that time. In addition, 95% received antibiotics, 18% corticosteroids and 1 patient received tocilizumab. None of the subjects received other antiviral agents (Table 2).

**Table 2:** Treatments Received at or after Enrollment

**Characteristic**	**Itraconazole (N=32)**	**Standard Care (N=33)**	**Total (N=65)**
Medication received during hospitalization			
Hydroxychloroquine – no. (%)	32 (100%)	32 (97%)	64 (99%)
Remdesivir – no. (%)	0 (0%)	0 (0%)	0 (0%)
Antibiotics – no. (%)	31 (97%)	31 (97%)	62 (95%)
Corticosteroids – no. (%)	6 (19%)	6 (18 %)	12 (19%)
Tocilizumab – no. (%)	0 (0%)	1 (3%)	1 (2%)
Admission to ICU – no. (%)	10 (31%)	9 (27%)	19 (29%)
Respiratory support			
Oxygen support – no. (%)	29 (91%)	29 (88%)	58 (89%)
High-flow oxygen – no. (%)	11 (34%)	8 (24%)	19 (29%)
Invasive mechanical ventilation – no. (%)	6 (19%)	5 (15%)	11 (17%)
ECMO – no. (%)	0 (0%)	0 (0%)	0 (0%)
Renal replacement therapy – no. (%)	1 (3%)	1 (3%)	2 (3%)

ICU denotes intensive care unit, ECMO extracorporeal membrane oxygenation

### 3.4 Efficacy Outcomes

**Figure 4:**
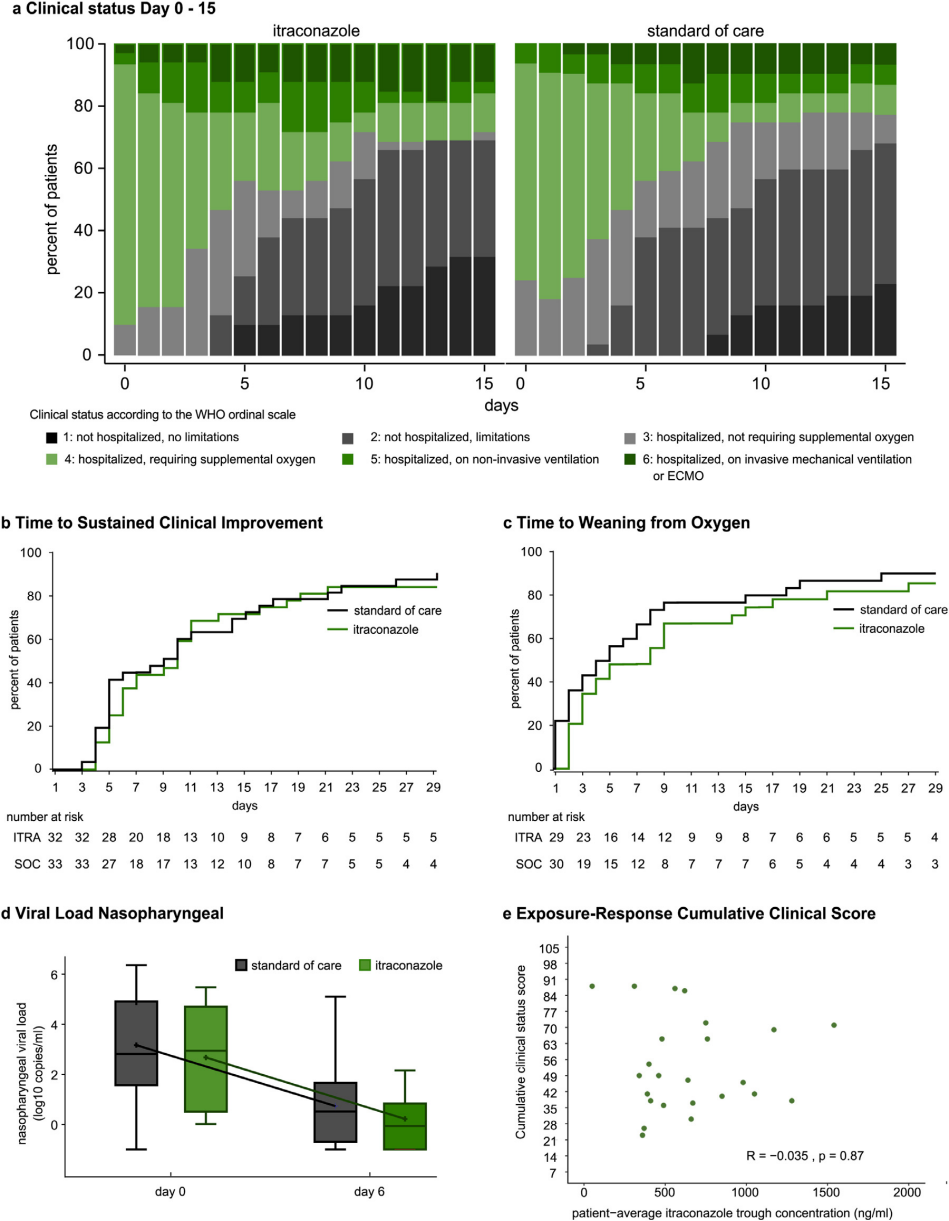
**Clinical outcomes. (a)** Bar chart of daily clinical status according to the WHO 7-point ordinal scale. **(b,c)** Cumulative estimates of **(b)** time to sustained clinical improvement **(c)** time to weaning from oxygen **(d)** Evolution of SARS-CoV-2 viral load from nasopharyngeal swabs assessed by RT-qPCR. Samples were available from 31 patients. Box plot shows median and interquartile range. Whiskers are drawn at (Q3 + 1.5 x IQR, Q 1- 1.5 x IQR). **(e)** Exposure-Response relation between itraconazole trough concentrations and cumulative clinical score day 1-15. Q1, Q3 denotes 1^st^ and 3^rd^ quartile, WHO denotes World Health Organization, IQR interquartile range.

Patients randomized to itraconazole or the standard of care group did not differ in cumulative clinical score at day 15. Mean (±SD): 49 (±20) *vs* 47 (±27) for itraconazole *vs* standard of care. Ratio of geometric mean (95% CI): 1.01 (0.85 to 1.19), p=0.91. (Table 3, Figure 4a). In addition, no differences were observed in time to sustained clinical improvement, in time to weaning from oxygen (Figure 4b, c) or in time to live discharge from the hospital (Supplemental Figure 2). The evolution of the NEWS score, as well as C-reactive protein were similar in both groups (Supplemental Figure 2). Nasopharyngeal swabs on day 6 ± 2 were obtained from 32 patients (47%). Viral load at baseline and on day 6 did not differ between study groups (Figure 4e) .

**Table 3:** Primary and Secondary Clinical Outcomes

**Outcome**	**Itraconazole (N=32)**	**Standard Care (N=33)**	**Treatment Effect**	**Estimate (95% CI)**
Cumulative status on day 15 – mean (SD)	49 (20)	47 (17)	Ratio of geometric means	1.01 (0.85 to 1.19)
Time to sustained clinical improvement - median [days] - median (IQR)	10 (5 to 18)	9 (5 to6)	Subdistribution HR	0.94 (0.56 to 1.60)
Day 28 mortality – no. (%)	0 (0%)	0 (0%)	HR	
Time to weaning from oxygen [days] – median (IQR) #	5 (2 to 15)	4 (1 to 8)	Subdistribution HR	0.76 (0.46 to 1.25)
Hospital stay [days] – median (IQR)	8 (4 to 17)	9 (4 to 16)	Subdistribution HR	0.92 (0.55 to 1.53)
ICU stay [days] – median (IQR) *	14 (8 to 22)	12 (9 to 18)	Subdistribution HR	0.76 (0.34 to 1.70)
Duration of mechanical ventilation – median (IQR) *	12 (8 to 16)	11 (7 to 12)	Subdistribution HR	0.37 (0.11 to 1.29)
Clinical Status on day 15– no. (%)			Common Odds Ratio	1.21 (0.47 to 2.87)
1: Not hospitalized, no limitations on activities	10 (31%)	7 (23%)		
2: Not hospitalized, limitations on activities	12 (38%)	14 (45%)		
3: Hospitalized, not requiring supplemental oxygen	1 (3%)	3 (10%)		
4: Hospitalized, requiring supplemental oxygen	4 (13%)	3 (10%)		
5: Hospitalized, on non-invasive ventilation or high flow oxygen devices	1 (3%)	2 (7%)		
6: Hospitalized, requiring ECMO, invasive mechanical ventilation, or both	4 (13%)	2 (7%)		

SD denotes standard deviation, IQR interquartile range, ECMO extracorporeal membrane oxygenation

# includes all patients: patients who did not receive supplemental oxygen were given a duration of 0 days

* Does not include patients who were not admitted to ICU/did not receive mechanical ventilation. Hence, the difference between treatments cannot be unambiguously be attributed to treatment

### 3.5 Safety Outcomes

There were no differences in ECG parameters, including QT-interval, between groups. Intensive care admission was required in 31 % in the itraconazole group vs 28 % in patients receiving standard of care. Beside the prespecified outcomes, few adverse events were reported as serious. No patients died during follow-up.

### 3.6 Pharmacokinetic analysis

Trough concentrations varied from 0.05 up to 1.94 mg/L for itraconazole and from 0.08 to 2.28 mg/L for hydroxy-itraconazole (Supplementary Table). Targets for itraconazole and hydroxy-itraconazole were attained in 37 and 21% of patients within 72h after initiation of treatment, which increased to 84 and 95% of patients during maintenance treatment, i.e. more than 72h after initiation.

Exposure-response analysis did not reveal a correlation between itraconazole trough concentrations and cumulative clinical status (Figure 4e), time to clinical improvement or decrease in nasopharyngeal viral load (Supplemental Figure 2).

## 4 Discussion

Despite antiviral activity of itraconazole in an *in vitro* antiviral assay against SARS-CoV-2, itraconazole is unlikely to be of clinical benefit in the treatment of COVID-19. The combined analysis of data from a preclinical hamster COVID-19 model [6,7 and a pilot clinical trial in hospitalized COVID-19 patients led to this conclusion.

This approach is unusual, as preclinical experiments normally precede clinical testing. However, the speed of the COVID-19 pandemic spread impeded the normal process of drug development. Because COVID-19 animal models were not available during the first months of the pandemic, drugs that were identified as *in vitro* inhibitors of SARS-CoV-2 were evaluated in clinical trials without preclinical evaluation in animals. Some, such as hydroxychloroquine or ivermectin, even became part of routine clinical care without any proof of their clinical efficacy [20].

When we discovered the *in vitro* antiviral activity of itraconazole we envisioned its potential use in the COVID-19 pandemic as the drug is widely available, affordable, accumulates well in lung tissue and has a well-known safety profile. On the other hand, we judged that the preclinical evaluation and a proof-of-concept clinical study was required before the potential anti-SARS-CoV-2 activity of itraconazole was broadly communicated. Like hydroxychloroquine, the antiviral activity of itraconazole is probably mediated via host-directed mechanisms. Itraconazole was reported to inhibit enterovirus replication by disrupting lipid transfer between the endoplasmic reticulum and the Golgi apparatus through inhibition of the oxysterol-binding protein [21]. A similar mechanism is probably at play for SARS-CoV-2. Although resistance is less likely for host-directed antivirals, these compounds are mostly less potent in terms of antiviral activity when compared with direct acting antivirals, such as those used to treat HIV, hepatitis C, influenza and herpesviruses.

We thus designed a pilot proof-of-concept clinical trial and did not communicate the *in vitro* findings to avoid a potential rush on the drug. As we anticipated that insights in the clinical management would evolve rapidly, we opted for a flexible trial design that is also used for the evaluation of other antiviral strategies [11], [12], [13].

After recruitment of 68 patients, an interim review of the pharmacokinetic data showed that the predefined range of itraconazole was reached at day 6, but not at day 2. The DSMB, therefore, recommended to halt the study, awaiting more pharmacokinetic and preclinical data. Meanwhile, we developed the COVID-19 hamster models that allowed to test the *in vivo* efficacy of itraconazole. As itraconazole failed to show any activity in these models, even when very high lung exposure was attained, we decided to discontinue the clinical trial, despite not having reached the foreseen 200 patients.

Even though the premature discontinuation of the study does not allow to draw firm conclusions, we have strong arguments that itraconazole is highly unlikely to be of any clinical use in the treatment of COVID-19. Firstly, itraconazole was not effective in both a treatment and transmission COVID-19 hamster model. In this model, we recently demonstrated the activity of high doses of the direct acting antiviral favipiravir, and the lack of efficacy of hydroxychloroquine [6]. Secondly, the data of our pilot clinical trial revealed no signals of a more positive outcome in any of the specified clinical endpoints in patients treated with itraconazole. Also, intermediate parameters such as decrease in viral load, oxygen demand, evolution of C-reactive protein or NEWS-score, failed to show any effect of itraconazole. In addition, there was a complete absence of an exposure response relationship between itraconazole trough levels and clinical outcomes.

A limitation of this study is the late presentation of hospitalized patients (median of 8 days after onset of symptoms), while antivirals are probably most beneficial when given early. Therefore, the potential benefit of itraconazole in early disease cannot be fully excluded. However, in light of the negative preclinical findings, we consider this rather unlikely and do not recommend the further development of itraconazole against COVID-19 for other disease stages or in other populations.

Our findings provide useful insights into the strategy of drug repurposing. In the absence of effective broad-spectrum antivirals, drug repurposing provides the only hope to rapidly identify inhibitors that may reduce viral replication in patients. However, caution is needed when introducing drugs into clinical care based solely on their *in vitro* activity. Because preclinical models are now available, molecules with promising *in vitro* antiviral activity against SARS-CoV-2 should undergo careful preclinical evaluation before planning clinical studies.

## Contributors

LLi, PeV, IS, BV, LvdL, TV, TD, GM, WJ, RV ,GV, JW, PDM, EVW drafted the clinical trial protocol and oversaw the study. LLi, IG, LAT, TG, ME, EL, VG, HC, BD were responsible for patient recruitment and follow-up. BV performed cardiac monitoring and safety analysis. IS, LVdL, ED and PiV conducted pharmacokinetic analysis. AB, GV performed statistical analysis. LLi, SJ, LLa, StH, RB, JDRP, SJFK, LD and JN conducted preclinical studies. JDRP, SJFK, LD, JN designed preclinical experiments. LLi, SJ, RB, StH, LLa performed preclinical experiments. NDTD, WC, XW and XZ performed the in vitro experiments. DJ and JN designed the in vitro experiments. LLi and PeV drafted the manuscript. All authors gave significant input on the manuscript and read and approved the final version. JN, EVW and PeV contributed equally

## Declaration of interests

Initial dug screening and discovery of the antiviral effect of itraconazole was done in collaboration with Johnson & Johnson, who also provided funding for this initial drug screen. Later in vitro drug screening was done independently from the company (e.g. antiviral activity of other azoles). Another manuscript in which an extensive panel of data is presented on the in vitro antiviral activity of itraconazole will be published together with authors from Johnson and Johnson. (Currently available as preprint [1]) Scientists from Johnson & Johnson performed drug measurements on hamster samples and provided guidance on the dosing regimens for the preclinical studies, but had no additional role in these experiments. The company had no role in the design, execution, analysis, publication or funding of the clinical trial. P Verhamme reports grants from KU Leuven during the conduct of the study and grants and personal fees for lectures and consultancy from Bayer Healthcare, Daiichi Sankyo, Pfizer, BMS, Bayer and Boehringer outside the submitted work, and personal fees for consultancy from Boehringer Ingelheim and Portola, outside the submitted work. Other authors have no conflict of interest to declare.

## Data sharing

The study protocol, statistical analysis plan, full statistical analysis informed consent form and clinical study report are provided in the in the Supplementary Material section. Anonymized raw data will be made available upon request according to GDPR regulation

## Appendix Supplementary materials

Image, application 1

Image, application 1

Image, application 1

Image, application 1

Image, application 1

Image, application 1
