# Supplementary material for: Itraconazole for COVID-19: preclinical studies and a proof-of-concept randomized clinical trial
Source: eBioMedicine. 2021 Mar 19;66:103288. doi: 10.1016/j.ebiom.2021.103288 (PMC7979145; doi:10.1016/j.ebiom.2021.103288)
Supplement: Supplementary file 1 [file mmc1.docx]

Supplementary Material

# Supplementary table

| Supplementary Table Overview of Itraconazole and hydroxy-itraconazole Exposure | | | | |
| --- | --- | --- | --- | --- |
|  |  | All patients | Patients treated with capsules | Patients treated with solution |
| Overall exposure | Number of patients with Ctrough monitoring – no. | 24 | 9 | 18 |
|  | Number of Ctrough samples – no. | 54 | 11 | 43 |
|  | Ctrough, itraconazole [mg/L] – median (IQR) | 0.50 (0.37 to 0.92) | 0.36 (0.22 to 0.62) | 0.65 (0.45 to 1.00) |
|  | Number of patients with Ctrough, itra > 0.5 mg/L – no. (%) | 18 (75%) | 4 (44%) | 15 (83%) |
|  | Ctrough, OH- itraconazole [mg/L] – median (IQR) | 0.89 (0.54 to 1.17) | 0.50 (0.38 to 0.94) | 0.94 (0.57 to 1.17) |
|  | Number of patients with Ctrough, OH-itra > 0.75 mg/L – no. (%) | 19 (79) | 4 (44) | 16 (89) |
| Early exposure | Number of patients with Ctrough monitoring – no. | 19 | 5 | 14 |
|  | Number of Ctrough samples – no. | 22 | 6 | 16 |
|  | Sampling day [days] – median (IQR) | 1.54 (1.44 to 1.71) | 1.53 (1.13 to 1.60) | 1.54 (1.48 to 1.73) |
|  | Ctrough, itraconazole [mg/L] – median (IQR) | 0.38 (0.24 to 0.69) | 0.25 (0.22 to 0.35) | 0.45 (0.33 to 0.77) |
|  | Number of patients with Ctrough, itra > 0.5 mg/L – no. (%) | 7 (37) | 1 (20) | 6 (43) |
|  | Ctrough, OH- itraconazole [mg/L] – median (IQR) | 0.54 (0.4 to 0.63) | 0.43 (0.37 to 0.49) | 0.58 (0.41 to 0.89) |
|  | Number of patients with Ctrough, OH-itra > 0.75 mg/L – no. (%) | 4 (21) | 0 (0) | 4 (29) |
| Late exposure | Number of patients with Ctrough monitoring – no. | 19 | 5 | 15 |
|  | Number of Ctrough samples – no. | 32 | 5 | 27 |
|  | Sampling day after start of treatment | 5.79 (5.5 to 8.65) | 5.86 (5.53 to 9.20) | 5.75 (5.5 to 7.99) |
|  | Ctrough, itraconazole [mg/L] – median (IQR) | 0.66 (0.54 to 1.05) | 0.57 (0.36 to 0.67) | 0.69 (0.54 to 1.05) |
|  | Number of patients with Ctrough, itra > 0.5 mg/L – no. (%) | 16 (84) | 3 (60) | 14 (93) |
|  | Ctrough, OH- itraconazole [mg/L] – median (IQR) | 1.08 (0.76 to 1.33) | 1.11 (0.77 to 1.23) | 1.08 (0.84 to 1.34) |
|  | Number of patients with Ctrough, OH-itra > 0.75 mg/L – no. (%) | 18 (95) | 4 (80) | 15 (100) |

# Supplementary figures

**Supplementary Figure 1: Additional Preclinical Data. (A)** Weight evolution of hamsters in the acute infection model, expressed as % weight change compared to day 0. Bars represent mean ± standard deviation. (B) Relationship between lung tissue itraconazole trough levels (C_trough)_ and lung viral load assessed by RT-qPCR in hamsters treated with itraconazole 70 mg/kg/day in the acute infection model. (C) Evolution of viral RNA levels from nasal swabs of hamsters in the acute infection model, quantified by RT-qPCR. Bar represents median.

**Supplementary Figure 2: Additional Clinical Data. (A)** Evolution of C-reactive protein and **(B)** evolution of NEWS score in trial participants. Box plot shows median and interquartile range. Whiskers are drawn at (Q3 + 1.5 x IQR, Q1- 1.5 x IQR). **(C)** Cumulative estimates of time to sustained clinical improvement **(D)** Exposure-Response relation between itraconazole trough concentrations and time to clinical improvement. **(E)** Exposure-Response relation between itraconazole trough concentrations and decrease in nasopharyngeal viral load. NEWS denotes National Early Warning Score, Q1, Q3 1^st^ and 3^rd^ quartile, IQR interquartile range

# Supplementary methods

## SARS-CoV-2 strain

The BetaCov/Belgium/GHB-03021/2020 (EPI ISL 407976|2020-02-03) strain, was recovered from a nasopharyngeal swab taken from a patient returning from Wuhan, China in February 2020. For these experiments a passage 6 virus, obtained after serial passaging on HuH7 and Vero E6 cells16 was used. Experiments were conducted in the high-containment A3 and BSL3+ facilities of the KU Leuven, Rega Institute (3CAPS) under licenses AMV 30112018 SBB 219 2018 0892 and AMV 23102017 SBB 219 20170589 according to institutional guidelines.

## Cells

Vero E6 cells (African green monkey kidney, ATCC CRL-1586) were cultured in minimal essential medium (Gibco) supplemented with 10% fetal bovine serum (Integro), 1% L-glutamine (Gibco) and 1% bicarbonate (Gibco). End-point titrations were performed with medium containing 2% fetal bovine serum instead of 10%. We calculated TCID_50_ using the Reed and Muench method. VeroE6-EGFP cells were kindly provided by Janssen Pharmaceutica and cultured in DMEM (Gibco) supplemented with 10% v/v heat-inactivated FCS and sodium bicarbonate (Gibco).

## *In vitro* antiviral assay

In this assay the eGFP positive cell culture shows a decrease in fluorescence upon infection by SARS-CoV-2 due to a massive cytopathogenic effect (CPE). In the presence of a selective antiviral the CPE is inhibited and the fluorescent eGFP signal is maintained. In brief the assays were performed as follows. On day -1, the test compounds were serially diluted in assay medium (same as culture medium but with 6% v/v heat-inactivated FCS). Diluted compounds were then mixed with VeroE6-eGFP cells at 3000 cells/well in 96-well plates (Greiner Bio-One). The plates were incubated overnight in a humidified incubator at 37°C and 5% CO2. On day 0, SARS-CoV-2 was added at 20 TCID50/well and on day 5 p.i. the eGFP fluorescence was determined using microscopy.

## Animals

Female Syrian gold hamsters, 6-10 weeks of age were purchased from Janvier Laboratories. The institutional Ethical Committee approved all animal experiments (license P065-2020).

## Histology

Lungs were fixed 4% formaldehyde and later embedded in paraffin. Tissue sections (5 μm) were stained hematoxylin and eosin and scored blindly for lung damage by a pathologist. The scored consisted of 10 parameters, to which a score of 0 to 3 was attributed; congestion, intralveolar hemorrhagic, apoptotic bodies in bronchus wall, necrotizing bronchiolitis, perivascular edema, bronchopneumonia, perivascular inflammation, peribronchial inflammation and vasculitis.

## SARS-CoV-2 RT-qPCR and end-point virus titrations on hamster tissue

Hamsters tissues were homogenized with bead disruption (Precellys) in 350 µL RLT buffer (RNeasy Mini kit, Qiagen) and RNA was extracted according to the manufacturer’s instructions. RT-qPCR was performed on a LightCycler96 platform (Roche) with iTaq Universal Probes One-Step RT-qPCR kit (BioRad) with N2 primers and probes targeting the nucleocapsid16. Standards of SARS-CoV-2 cDNA (IDT) were used to express viral genome copies per mg tissue. Forward primer: 5’-TTA CAA ACA TTG GCC GCA AA-3’. Reverse primer: 5’-GCG CGA CAT TCC GAA GAA-3’ . Probe: 5’-FAM-ACA ATT TGC CCC CAG CGC TTC AG-BHQ1-3’.

For end-point titrations, lung tissue was homogenized in 350 µL minimal essential medium and endpoint titrations were performed on confluent Vero E6 cells in 96-well plates. Viral titers were calculated by the Reed and Muench method and expressed as 50% tissue culture infectious dose (TCID_50_) per mg tissue.

## SARS-CoV-2 RT-qPCR of patient samples

Subjects underwent nasopharyngeal swabs, which were placed in 5 mL Universal Transport Medium (UTM). Virus in 100 µL of the UTM was lysed by adding 900 µL TRIzol™ Reagent (Invitrogen) and an internal control of Phocine morbillivirus (PDV). Total RNA was isolated by adding 200 µL of chloroform, followed by centrifugation and isolation of the clear aqueous phase. The RNA was precipitated using Glycoblue™ in isopropanol and centrifugation. The RNA pellet was washed with 75% ethanol before suspending it in 30 µL RNase free water. SARS-CoV-2 in 4 µL was detected with RT-qPCR on a Quant Studio 5 platform (Thermo Fisher) using Takyon One-step Low ROX probe 5X Mastermix dTTP, E_Sarbeco primers and E_Sarbeco FAM-probe (Charité protocol, National Reference Center for Respiratory Viruses, Institut Pasteur, Paris). Standards of SARS-CoV-2 cDNA (IDT) were used to express viral genome copies per mL. Viral titres were calculated by the Reed and Muench method. In parallel RT-qPCR was performed against the PDV internal control using PDV primers (forward: 5'-GGT GGG TGC CTT TTA CAA GAA C-3', reverse: 5'-ATC TTC TTT CCT CAA CCT CGT CC-3') and a PDV FAM-probe (5’(HEX)-ATGCAAGGG/ZEN/CCAATTCTTCCAAGTT ATCACTT-(3IABkFQ)3’).

# DAWn-Studies: Collaborators

The DAWn study team is grateful to all patients, their family members and all health care workers who helped to perform clinical studies in challenging times during the COVID-19 pandemic.

| Steering Committee DAWn-studies | |
| --- | --- |
| Eric Van Wijngaerden | Coordinating Investigator D-Itraco |
| Wim Janssens | Coordinating Investigator D-Azithro |
| Thomas Vanassche | Coordinating Investigator D-Antico |
| Geert Meyfroidt | Coordinating Investigator D-Plasma |
| Peter Verhamme | DAWn-RCT coordinator |
| Laurens Liesenborghs | Principal investigator D-Itraco |
| Robin Vos | Principal investigator D-Azithro |
| Timothy Devos | Principal investigator D-Plasma |
| Jan Gunst | D-Antico investigator |
| Joost Wauters | D-Antico and D-Itraco investigator |
| Paul De Munter | D- Itraco investigator |
| Johan Neyts | D-Itraco investigator / virologist |
| Carine Wouters | D-Antico investigator |
| Steffen Rex | D-Antico investigator |
| Lieven Dupont | D-Azithro investigator |
| Isabel Spriet | Expert pharmacologist |
| Geert Verbeke | Senior study statistician |
| Kathleen Claes | Clinical Operational Director, on behalf of sponsor |
| Wim Robberecht | CEO UZ Leuven, on behalf of sponsor |
| Chris Van Geet | Vice-Rector Biomedical sciences, on behalf of sponsor |
|  |  |
| Advisory Committee | |
| Willy Peetermans | Chairman Internal Medicine |
| Stefan Janssens | Cardiovascular Diseases |
| Greet Van den Berghe | Intensive Care Medicine |
| Katrien Lagrou | Laboratory Medicine |
| Peter Vandenberghe | Haematology |
| Geert Verleden | Respiratory Diseases |
| Dirk Kuypers | Nephrology |
| Sandra Verelst | Emergency Medicine |
| Marc Van de Velde | Anesthesiology |
| Marc Van Ranst | Laboratory Medicine |
|  |  |
| Study Coordinators DAWn-studies | |
| Barbara Debaveye | Clinical Trial Coordinator |
| Helga Ceunen | Clinical Trial Coordinator |
| Veerle Servaes | Study Coordinator D-Antico |
| Anna Ockerman | Study Coordinator D-Antico |
| Caroline Devooght | Study Coordinator D-Antico |
| Maylorie 't Lam | Study Coordinator D-Azithro |
| Kaat Haesendock | Study Coordinator D-Azithro |
| Myriam Cleeren | Study Coordinator D-Plasma |
| Jill Pannecoucke | Study Coordinator D-Plasma |
| Elisabeth Porcher | Study Coordinator D-Plasma |
| Katrien Cludts | Study Coordinator |
| Evelyn Marcelis | Study Coordinator |
| Annemie Devroye | Study Coordinator |
| Sophie Achten | Study Coordinator |
|  |  |
| Leuven Coordinating Centre (LCC) | |
| Rik Hendrickx |  |
| Anne Luyten |  |
| Katleen Vandenberghe |  |
| Peter Van Rompaey |  |
|  |  |
| COVID-19 Clinical Coordinators UZ Leuven | |
| Willy Peetermans | Department of General Internal Medicine |
| Alexander Wilmer | Department of General Internal Medicine, Medical Intensive Care |
| Tom Adriaenssens | Department of Cardiovascular Diseases |
| Stefanie Vandervelden | Department of Emergency Medicine |
| Philippe Dewolf | Department of Emergency Medicine |
| Marijke Peetermans | Department of General Internal Medicine, Medical Intensive Care |
| Philippe Meersseman | Department of General Internal Medicine, Medical Intensive Care |
| Christophe Vandenbriele | Department of Cardiovascular Diseases |
| Liesbeth Henckaerts | Department of General Internal Medicine |
| Greet Hermans | Department of General Internal Medicine, Medical Intensive Care |
| Peter Vanbrabant | Department of General Internal Medicine |
| Natalie Lorent | Department of Respiratory Diseases |
| Laurent Godinas | Department of Respiratory Diseases |
| Pascal Van Bleyenbergh | Department of Respiratory Diseases |
| Els Wauters | Department of Respiratory Diseases |
| Marion Delcroix | Department of Respiratory Diseases |
| Dieter Dauwe | Department of Intensive Care Medicine |
| Michaël Casaer | Department of Intensive Care Medicine |
| Yves Debaveye | Department of Intensive Care Medicine |
| Lars Desmet | Department of Intensive Care Medicine |
| Erwin De Troy | Department of Intensive Care Medicine |
| Greet De Vlieger | Department of Intensive Care Medicine |
| Greta Van den Berghe | Department of Intensive Care Medicine |
| Renata Haghedooren | Department of Intensive Care Medicine |
| Catherine Ingels | Department of Intensive Care Medicine |
| Bart Jacobs | Department of Intensive Care Medicine |
| Jan Muller | Department of Intensive Care Medicine |
| Dirk Vlasselaers | Department of Intensive Care Medicine |
| Marc Van de Velde | Department of Anesthesiology |
| Johan De Coster | Department of Anesthesiology |
| An Schrijvers | Department of Anesthesiology |
| Veerle De Sloovere | Department of Anesthesiology |
| Arne Neyrinck | Department of Anesthesiology |
| Astrid Barbé | Department of Anesthesiology |
| Steve Coppens | Department of Anesthesiology |
| Raf Van den Eynde | Department of Anesthesiology |
| Katleen Fagard | Department of Geriatric Medicine |
| Evelien Gielen | Department of Geriatric Medicine |
| João Pedro Guedelha Sabino | Department of Gastroenterology |
|  |  |
| Laboratory Medicine UZ Leuven | |
| Pieter Vermeersch |  |
| Katrien Lagrou |  |
| Ann Verdonck |  |
| Koen Poesen |  |
| Marc Jacquemin |  |
| Soumia Arredouani |  |
|  |  |
| Radiology Department | |
| Adriana Dubbeldam |  |
| Walter De Wever |  |
|  |  |
| Coordinating Investigator Medical Support Team | |
| Iwein Gyselinck |  |
| Matthias Engelen |  |
| Laure-Anne Teuwen |  |
| Tatjana Geukens |  |
| Vincent Geldhof |  |
| Quentin Van Thillo |  |
| Ewout Landeloos |  |
|  |  |
| Yannick Van Herck |  |
| Anke Van Herck |  |
| Pierre Van Mol |  |
|  |  |
| Pharmacy Safety Monitoring | |
| Isabel Spriet |  |
| Lorenz Van der Linden |  |
| Charlotte Quintens |  |
| Greet Van de Sijpe |  |
| Ruth Van Daele |  |
| Matthias Gijsen |  |
|  |  |
| ECG safety monitoring | |
| Bert Vandenberk | Department of Cardiovascular Medicine |
| Rik Willems | Department of Cardiovascular Medicine |
|  |  |
| [Leuven Biostatistics and Statistical Bioinformatics Centre (L-BioStat)](https://www.kuleuven.be/wieiswie/en/unit/50000696) | |
| Geert Verbeke |  |
| Ann Belmans |  |
| Kris Bogaerts |  |
|  |  |
| Data Safety Monitoring Board | |
| Séverine Vermeire | Chair |
| Emmanuel Lesaffre | Statistician |
| Joris Ector | Cardiologist |
| Jan de Hoon | Pharmacologist |
| Patrick Verschueren | Rheumatologist |
| Heidi Sterckx | Secretary |
|  |  |
| Clinical Trial Center (CTC) UZ Leuven | |
| Hilde De Tollenaere |  |
| Peter Van Rompaey |  |
| Klara Vlassak |  |
| Heidi Sterckx |  |
| Katrien Boulanger |  |
| Jean-Jacques Derèze |  |
|  |  |
| Other Support | |
| Diane De Wyngaert | Administrative support |
| Wouter Cypers | IT |
| Jurgen Silence | IT |
| Alexander Otten | IT |
| Kevin Vits | IT |
| Ruth Storme | Ethical Committee |
| Kathleen Schuyten | Administrative support |
| Nadine Ectors | Biobank |
| Loes Linsen | Biobank |
| Dirk Jochmans | REGA |
| Inge Wullaert | KU Leuven fundraising |
| Nadine Loenders | KU Leuven fundraising |
| Kristine Chapelle | KU Leuven fundraising |
| Bas Aerts | ICU nurse |
|  |  |
| All Health Care Workers of all COVID-19 units UZ Leuven | |

Table 1. Collaborators of the DAWn studies. D-Itraco = DAWn-Itraconazole; D-Azithro= DAWn-Azithromycine; D-Antico = DAWn-Antico; D-Plasma = DAWn-Plasma; D-RCT = DAWn-Randomized Controlled Trials, i.e. D-Itraco, D-Azithro, D-Antico and D-Plasma;
