# Supplementary material for: Itraconazole for COVID-19: preclinical studies and a proof-of-concept randomized clinical trial
Source: eBioMedicine. 2021 Mar 19;66:103288. doi: 10.1016/j.ebiom.2021.103288 (PMC7979145; doi:10.1016/j.ebiom.2021.103288)
Supplement: Supplementary file 2 [file mmc2.pdf]

CLINICAL TRIAL PROTOCOL

A randomized, open-label, adaptive, proof-of-concept clinical trial of new antiviral drug candidates against SARS-CoV-2

Direct antivirals working against nCoV (DAWN trial)

Version number: v 1.0 – **Date** 24/03/2020

EudraCT Nbr: 2020-001243-15

Sponsor

University Hospitals Leuven (UZ Leuven)

Herestraat 49, B-3000 Leuven

Coordinating Investigator

Prof Dr Eric Van Wijngaerden

Confidentiality Statement

The information in this document is strictly confidential and is available for review to Investigators, potential Investigators and appropriate Ethics Committees, Institutional Review Boards or Competent Authorities. No disclosure should take place without written authorization from the Sponsor.

CLINICAL TRIAL PROTOCOL HISTORY

CTP / Amendment #	Date	Reason for amendment
Clinical Trial Protocol v1	24/03/2020	NA
Clinical Trial Protocol v2	24/03/2020	Specification of dosing and duration of IMP

LIST OF PARTICIPATING SITES

(as applicable)

List Of Participating Sites	Principal Investigator
UZLeuven	Prof. Dr. Eric Van Wijngaerden

SIGNATURES

Title: A randomized, open-label, adaptive, proof-of-concept clinical trial of new antiviral drug candidates against SARS-CoV-2

Protocol: Direct antivirals working against nCoV (DAWN trial)

The undersigned confirm that the above referenced protocol has been acknowledged and accepted, and agree to conduct the Trial in compliance with the approved protocol, and will adhere to: the principles outlined in the requirements for the conduct of clinical trials in the EU as provided for in Directive 2001/20/EC or the EU Regulation 536/2014 (as soon as in effect) and any subsequent amendments thereto, the ICH guidelines, the most recent version of the Declaration of Helsinki, the Belgian law of May 7th 2004 regarding experiments on the human person (as amended) or the Belgian law of May 7th 2017 related to clinical trials on medicinal products for human use (as soon as in effect), the EU General Data Protection Regulation 2016/679 (GDPR), the relevant Belgian laws implementing the GDPR, the Belgian Law of August 22nd 2002 on patient rights, the Sponsor's applicable SOPs, and other regulatory requirements as applicable.

The undersigned agree not to disclose the confidential information

contained in this document for any purpose other than the evaluation or conduct of the Trial, without prior written consent of the Sponsor.

The undersigned also commit to making the findings of the Trial publicly available through publication and/or other dissemination tools, in accordance with this protocol and applicable regulations, without any unnecessary delay and to provide an honest, accurate and transparent account of the Trial; and to explain any discrepancies or deviations from the approved Trial protocol.

Coordinating Investigator

Prof. Dr. Eric Van Wijngaerden	....	....
Name & Title	Signature	Date

Principal Investigator (Participating Site) *(in case of monocentric Trial, the Principal Investigator is the same as the Coordinating Investigator)*

Prof. Dr. Eric Van Wijngaerden	....	....
Name & Title	Signature	Date

TABLE OF CONTENTS

CLINICAL TRIAL PROTOCOL 1
CLINICAL TRIAL PROTOCOL HISTORY 2
LIST OF PARTICIPATING SITES 2
SIGNATURES 3
TABLE OF CONTENTS 2
LIST OF ABBREVIATIONS 5
FUNDING AND SUPPORT 9
ROLES AND RESPONSIBILITIES 9
TRIAL SYNOPSIS 10
TRIAL FLOWCHART 12
1 Background and Rationale 14
2 Trial Objectives and Design 15
2.1 Trial objectives 15
2.2 Trial outcomes 16
2.2.1 Primary outcome 16
2.2.2 Secondary outcome 16
2.2.3 Exploratory outcomes 16
2.3 Trial Design 17
2.4 Expected Duration of the Trial 17
3 Trial Population / Eligibility Criteria 18
3.1 Inclusion criteria 18
3.2 Exclusion criteria 18
4 Trial Procedures 19
4.1 Participant Consent and withdrawal of consent 19
4.2 Selection of Participants / Recruitment 19
4.3 Randomization Procedure 19
4.4 Trial Procedures 19
4.4.1 By visit 19
4.4.2 Laboratory tests 20
4.4.3 Other investigations 20
4.5 Premature discontinuation of Trial treatment 20
5 Trial Medication / Drug 22
5.1 Investigational Medicinal Product and Dosing Regimen 22
5.2 Concomitant / Prohibited Medication / Treatment 22
6 Safety 23

6.1 Specification, timing and recording of safety parameters 23
6.2 Specification, timing and recording of safety parameters 23
6.2.1 Definitions 23
6.2.2 Adverse Events that do not require reporting 23
6.2.3 Recording and reporting of Adverse Events 24
6.2.4 Assessment 24
6.2.5 Timelines for reporting 25
6.2.6 Follow-up 25
6.2.7 Death 25
6.2.8 Reporting requirements to Ethics Committee's (EC's) and Competent Authorities (CA's) 25
6.2.9 Sponsor's reporting of Suspected Unexpected Serious Adverse Reactions (=SUSARs) 26
6.2.10 Annual reporting 26
6.2.11 8.4 Data monitoring committee (DMC) and Treatment stopping rules 26
7 Statistics and Data Analysis 27
7.1 Sample Size Determination 28
7.2 Statistical Analysis 28
7.3 Trial Steering Committee (TSC) / Data Monitoring Committee (iDMC) 30
8 Data handling 31
8.1 Data Collection Tools and Source Document Identification 31
8.1.2 Legal requirements 31
8.2 Audits and Inspections 32
8.3 Monitoring 32
8.4 Archiving 32
9 Ethical and Regulatory Considerations 33
9.1 Ethics Committee (EC) review & reports 33
9.2 Regulatory Compliance 33
9.3 Protocol / GCP compliance 33
9.4 Data protection and participant confidentiality 33
9.5 Insurance 34
9.6 Amendments 34
9.7 Post-Trial activities 34
10 Research Registration, Dissemination of Results and Publication Policy 34
11 Intellectual Property 35
12 Joint Commission International (JCI) 35
References 36
APPENDICES 37
13 Appendix 1: Data Processing Annex (DPA) (in case the study will become multicentric) 37
14 Appendix 2: Drug-Drug Interactions Itraconazole 39

LIST OF ABBREVIATIONS

Abbreviation	Definition
(e)CRF	(electronic) Case Report Form
AE	Adverse Event
AESI	Adverse Event of Special Interest
APR	Annual Progress Report
ASR	Annual Safety Report
AR	Adverse Reaction
CA	Competent Authority
CI	Coordinating Investigator
CIOMS	Council for International Organizations of Medical Sciences
CM	Concomitant Medication
CSR	Clinical Study Report
CTP	Clinical Trial Protocol
DMC	Data Monitoring Committee
DMP	Data Management Plan
DPA	Data Processing Annex
DSMB	Data Safety Monitoring Board
DSUR	Development Safety Update Report
EC	Ethics Committee
ECG	Electrocardiogram
EoT	End of Trial
FPFV	First Patient First Visit
GCP	Good Clinical Practice (latest version of ICH E6)
IB	Investigator's Brochure
ICF	Informed Consent Form
ICH	International Conference on Harmonisation
IMP	Investigational Medicinal Product
ISF	Investigator Site File
LPLV	Last Patient Last Visit
MAH	Marketing Authorisation Holder
MP	Monitoring Plan
PI	Principal Investigator (Participating Site)
PRO	Patient Reported Outcome
SAE	Serious Adverse Event
SAP	Statistical Analysis Plan
SAR	Serious Adverse Reaction
SmPC	Summary of Product Characteristics
SUSAR	Suspected Unexpected Serious Adverse Reaction
TMF	Trial Master File

TSC	Trial Steering Committee

FUNDING AND SUPPORT

Funder	Type of Financial or Non-Financial Support
UZ Leuven	Financial and Non-Financial Support

No fault liability insurance has been taken out by UZ Leuven for treating and/or compensating Trial participants who are harmed as a consequence of participation in the Trial.

ROLES AND RESPONSIBILITIES

The Principle Investigator (PI) is responsible for the conduct of the Trial at his/her Participating Site, and for protecting the rights, safety and well-being of the Trial participants. As such the PI must ensure adequate supervision of the Trial conduct at the Participating Site. If any tasks are delegated, the PI will maintain a log of appropriately qualified persons to whom he/she has delegated specified Trial-related duties. The PI will ensure that adequate training is provided and documented for all Trial staff, prior to conducting assigned Trial-related activities.

It is the Coordinating Investigator's (CI's) responsibility to supervise the general conduct (e.g. Trial progress, communication, protocol training and support of the participating sites, annual reporting to the Ethics Committee (EC), end of Trial notification(s) and results reporting…) of the Trial. The CI fulfils both Investigator and Sponsor responsibilities, as outlined in International Conference on Harmonisation – Good Clinical Practice (ICH-GCP) E6(R2) and applicable regulations.

PI and CI shall each be referred to as « Investigator(s)».

TRIAL SYNOPSIS

Title of clinical Trial («Trial»)	A randomized, open-label, adaptive, proof-of-concept clinical trial of new antiviral drug candidates against SARS-CoV-2
Protocol Short Title Acronym	Direct antivirals working against nCoV (DAWN trial)
Trial Phase (I, II, III, IV)	phase II proof-of-concept study
Sponsor name	University Hospitals Leuven (UZ Leuven)>
Coordinating Investigator	Prof Dr Eric Van Wijngaerden
Contact Address CI	University Hospitals Leuven – Internal Medicine
Contact Email CI	Eric.VanWijngaerden@uzleuven.be
Contact Phone CI	+ 32 16 34 47 75
Trial permancy /emergency contact	Dr. Laurens Liesenborghs – Rega Institute
Contact email	Laurens.liesenborghs@kuleuven.be
Contact phone	+32 16 36 26 or +32 475 31 19 04
EudraCT number	2020-001243-15
Other public database nbr	/
Principal Investigators and Participating Sites	University Hospitals Leuven (UZ Leuven)
Medical condition or disease under investigation	COVID-19
Trial rationale	To evaluate clinical efficacy and safety of investigational therapeutics for COVID-19
Primary objective	The overall objective of the study is to evaluate the clinical efficacy and safety of different investigational therapeutics relative to the standard of care in patients hospitalized with COVID-19.
Secondary objective(s)	To evaluate clinical efficacy of different investigational therapeutics as compared to the control arm as assessed by Clinical Severity, Oxygenation, Ventilation, Hospitalisation.
Trial Design	Randomized, Open-label, Multicentre, Adaptive Study design
Outcomes	Clinical status of subject until day 15 (on a 7-point ordinal scale):<br>1. Not hospitalized, no limitations on activities<br>2. Not hospitalized, limitation on activities;<br>3. Hospitalized, not requiring supplemental oxygen;<br>4. Hospitalized, requiring supplemental oxygen;<br>5. Hospitalized, on non-invasive ventilation or high flow oxygen devices;<br>6. Hospitalized, on invasive mechanical ventilation or ECMO;<br>7. Death.<br>Exploratory secondary outcomes
Sample Size	200
IMP, dosage and route of administration	Itraconazole, LD on day 1-3: 3 x 200 mg, MD: 2 x 200 mg from day 4-15, PO

	Other investigational products may be added as part of the adaptive study design
Active comparator product(s)	none
Maximum duration of treatment and Follow Up of a Participant	15 days of treatment<br>30 days of follow-up
Maximum duration of entire Trial	3 years
Date anticipated First Participant First Visit (FPFV)	25 March 2020
Date anticipated Last Patient Last Visit (LPLV)	unknown

TRIAL FLOWCHART

Schedule of Events – Trial specific Procedures / Assessments

	**Screen**	**Baseline**				
**Day +/- window**	**-1 or 1**	**1**	**Daily until discharge**	**6 +/- 2**	**15 +/- 2**	**29 +- 3**
**Assesments/Procedures**						
**ELIGIBILITY**						
Informed consent	X					
Demographics & Medical History	X					
Review SARS-CoV-2 results	X					
In- and exclusion criteria	X					
**STUDY INTERVENTION**						
Randomization		X				
Administration of study drug		X	Daily for at least 10 days and up to Day 15 or discharge			
**STUDY PROCEDURES**						
Vital signs including SpO2		X	Daily until discharge			
Clinical data collection		X	Daily until discharge			
Targeted medication review		X	Daily until discharge			
Adverse event evaluation		X	Daily until discharge			
ECG		X	QT-monitoring scheme			
Evaluation by telephone					X	X

**LABORATORY**						
CRP, haematology, chemistry, kidney and liver test	X	At clinician's discretion	At clinician's discretion			
Pregnancy test for females of childbearing potential	X					
Viral qPCR (Nasopharyngeal swab)				If feasible		
itraconazole trough level				If feasible		

1 Background and Rationale

In December 2019, the Wuhan Municipal Health Committee identified an outbreak of viral pneumonia cases of unknown cause. Coronavirus RNA was quickly identified in some of these patients. This novel coronavirus has been designated SARS-CoV-2, and the disease caused by this virus has been designated COVID-19. Currently there are no approved therapeutic agents available for coronaviruses [1].

The aim of this study protocol is to investigate promising drug compounds in a proof-of-concept study. The design is adaptive, i.e. it allows to add and remove treatment arms and drug candidates based on the most updated information. The study complies with the recommendations for outcomes as outlined by the WHO master template protocol (https://www.who.int/emergencies/diseases/novel-coronavirus-2019/technical-guidance/early-investigations; and https://www.who.int/emergencies/diseases/novel-coronavirus-2019/global-research-on-novel-coronavirus-2019-ncov assessed on March 20th 2020).

In the Laboratory of Virology and Chemotherapy at the Rega Institute (KU Leuven), a library of existing drugs that were previously tested in clinical trials, of which some are available on the marked, was screened for activity against SARS-CoV-2 with the aim to repurposing drugs for COVID-19. It is expected that several compounds will be identified that show anti-viral activity in vitro, which subsequently need to be investigated in patients [2].

The first candidate drug with in vitro antiviral activity against SARS-CoV-2 is itraconazole. Itraconazole showed *in vitro* antiviral activity in the high throughput screening. Also computational modeling of drugs with potential anti-viral activity identified itraconazole as a drug candidate against SARS-CoV-2 [3]. Furthermore, itraconazole is active against feline corona species [4]. In addition, itraconazole was previously shown to inhibit Influenza A both in in vitro and in vivo models [5]. Itraconazol is a marketed drug, and is available on prescription in most countries (Market Access since 1988 in Belgium), and many generic preparations are available.

Eligible adult patients who tested positive for SARS-CoV-2 and are admitted to the hospital will be randomized and assessed daily during hospitalization. Discharged patients will be contacted by telephone at days 15 and 29. All subjects will undergo efficacy and safety assessments, including laboratory assays, which are aligned with clinical care. Also blood samples and nasopharyngeal swabs will be done according to clinical need (standard of care). If feasible an additional nasopharyngeal swab will be taken on day 7. The study should not put an extra burden on healthcare workers and on the hospital's resources.

2 Trial Objectives and Design

2.1 Trial objectives

The study objectives adapted from the WHO master protocol. (https://www.who.int/emergencies/diseases/novel-coronavirus-2019/technical-guidance/early-investigations; https://www.who.int/emergencies/diseases/novel-coronavirus-2019/global-research-on-novel-coronavirus-2019-ncov assessed on March 20th 2020).

The overall objective of the study is to evaluate the clinical efficacy and safety of investigational therapeutic agents relative to the standard of care in patients hospitalized with COVID-19.

Secondary objectives are to evaluate clinical efficacy of different investigational therapeutics as compared to the control arm as assessed by:

Clinical Severity

Ordinal scale:

- Time to an improvement of one category from admission on an ordinal scale.
- Subject clinical status on an ordinal scale at days 3, 5, 8, 11, 15 and 29.
- Mean change in the ranking on an ordinal scale from baseline to days 3, 5, 8, 11, 15 and 29 from baseline.

National Early Warning Score (NEWS):

- The time to discharge or to a NEWS of ≤ 2 and maintained for 24 hours, whichever occurs first.
- Change from baseline to days 3, 5, 8, 11, 15, and 29 in NEWS.

Oxygenation:

- Oxygenation free days in the first 28 days (to day 29).
- Incidence and duration of new oxygen use during the trial.

Mechanical Ventilation:

- Ventilator free days in the first 28 days (to day 29).
- Incidence and duration of new mechanical ventilation use during the trial.

Hospitalization

- Duration of hospitalization (days).

Mortality

- 15-day mortality
- 28-day mortality

Evaluate the safety of the intervention through 28 days of follow-up as compared to the control arm as assessed by:

- Cumulative incidence of serious adverse events (SAEs) and adverse events (AEs) graded as severe.
- Discontinuation or temporary suspension of drug administration (for any reason).
- Changes in white cell count, haemoglobin, platelets, creatinine, glucose, total bilirubin, ALT, and AST over time.

2.2 Trial outcomes

The study outcomes are based on the WHO master protocol. All outcomes will be presented overall as well as separately for patients with mild/moderate vs severe disease at baseline.

2.2.1 Primary outcome

Clinical status of subject at day 15 (on a 7-point ordinal scale):

1. Not hospitalized, no limitations on activities
2. Not hospitalized, limitation on activities;
3. Hospitalized, not requiring supplemental oxygen;
4. Hospitalized, requiring supplemental oxygen;
5. Hospitalized, on non-invasive ventilation or high flow oxygen devices;
6. Hospitalized, on invasive mechanical ventilation or ECMO;
7. Death.

Cumulative clinical status of subject up to day 15 (on a 7-point ordinal scale):
The sum of the daily clinical status score for days 1 up to 15.
Theoretical minimum is 17 (best-case scenario, discharge immediately after enrollment) and theoretical maximum is 105 (death on first study day).

2.2.2 Secondary outcome

- Status on an ordinal scale assessed daily while hospitalized and on days 15 and 29.
- Mortality on day 15 and day 30, time to death
- Time to clinical improvement (n° days from hospitalization to first 2-point improvement from highest previously recorded clinical state on the 7-point ordinal scale)
- Duration of supplemental oxygen (if applicable).
- Duration of mechanical ventilation (if applicable).
- Duration of hospitalization (separately for survivors and non-survivors)
- Duration of intensive care stay (separately for survivors and non-survivors)
- Date and cause of death (if applicable).
- NEWS assessed daily while hospitalized and on days 15 and 29.

- Adverse events graded as severe or SAEs.

- Lab values**:** CRP, white cell count, absolute neutrophil count, absolute lymphocyte count, haemoglobin, platelets, serumcreatinine, eGFR (CKD-EPI), hsTroponinT, glucose, potassium, total bilirubin, ALT, and AST on days 1; 3, 5, 8, 11, 15 and 29 (If measured according to clinical indication).

- Follow-up of absolute QTc and delta QTc interval between days 0-2, days 3-5, days 6-8, and days 9-10 of treatment on units with intermittent ECG monitoring or continuous follow-up on ICUs

- Combined cardiac endpoint (any of the following: hsTtroponinT levels >0.5ng/mL, ventricular arrhythmia requiring intervention, reanimation, sudden cardiac death)

2.2.3 Exploratory outcomes

- Qualitative and quantitative PCR for SARS-CoV-2 in (naopharyngeal) swab on day 1 and 6
- Itraconazole trough level on day 6

2.3 Trial Design

This study is an **adaptive, randomized, open-label clinical trial** to evaluate the safety and efficacy of promising antiviral agents in hospitalized adult patients diagnosed with COVID-19.
The outcomes of the study protocol are in part based on the draft master protocol of the WHO for trials that evaluate safety and efficacy of investigational therapeutics for the treatment of COVID-19 in hospitalized patients.
The study is a **phase 2 proof-of-concept multicenter trial**. It will be initiated as a single-center trial, though additional sites may be added during the course of the trial.
The study will compare standard of care vs. standard of care with the investigational therapeutic agent. Since there are no current approved treatment options for COVID-19, the standard of care is mostly supportive. However, the standard of care will reflect the guidance by (inter)national guidelines and hence may change during the course of the study.
The clinical outcomes of this study have been choosen based on the outcomes of the WHO master template for clinical studies to allow pooling of the data with other ongoing studies.

The adaptive study design allows for the addition of new treatment arms during the study by study amendment.

2.4 Expected Duration of the Trial

The trial is expected to start March 2020, with a duration of 3 years.

3 Trial Population / Eligibility Criteria

3.1 Inclusion criteria

Participants eligible for inclusion in this Trial must meet **all** of the following criteria:

1. Subject (≥18 years old) or legally authorized representative provides informed consent prior to initiation of any study procedures. When signed informed consent is not possible (e.g. due to restrictions to prevent viral transmission), verbal informed consent in the presence of a witness will be obtained and documented in the medical files. Signed informed consent will be obtained as soon as the safety concerns are mitigated.
2. Subject (or legally authorized representative) understands and agrees to comply with planned study procedures.
3. Male or non-pregnant female adult ≥18 years of age at time of enrolment.
4. Has laboratory-confirmed SARS-CoV-2 infection as determined by PCR, or other commercial or public health assay in any specimen as diagnosed within 72 hours prior to randomization.
5. Illness of any duration, and at least one of the following:
    a. Radiographic infiltrates by imaging (chest x-ray, CT scan, etc.), or
    b. Clinical assessment (evidence of rales/crackles on exam) AND SpO2 ≤ 94% on room air, or
    c. Requiring mechanical ventilation and/or supplemental oxygen.

All participants that are considered for Trial participation, per the above criteria will be documented on the Screening Log, including Screen Failures.

3.2 Exclusion criteria

Participants eligible for this Trial must **not** meet any of the following criteria:

1. ALT/AST > 8 times the upper limit of normal.
2. Pregnancy or breast feeding.
3. Allergy to any study medication.
4. Any medical condition which would impose an unacceptable safety hazard by participation to the study.
5. Study drug specific exclusion criteria:
    - for itraconazole:
        - heart failure with severely reduced ejection fraction (≤ 30%)
        - concomitant treatment with lopinavir/ritonavir and potent CYP450 inducers such as rifampicin, rifabutin, carbamazepine, phenobarbital, primidon, phenytoin, St-John's wort as active treatment or taken up to 10 days before randomization.

Participants who meet one or more of the above exclusion criteria **must not proceed** to be enrolled/randomized in the Trial and will be identified on the Screening Log as Screen Failure.

4 Trial Procedures

4.1 Participant Consent and withdrawal of consent

The Trial will be conducted only on the basis of prior informed consent by the Trial participants and/or their legally authorized representative(s). As such, no Trial-related procedures will be conducted prior to obtaining written informed consent from potential Trial participants.

When signed informed consent is not permitted because of safety regulations related to the prevention of the transmission of SARS-CoV-2, verbal informed consent shall be documented in the medical records. Signed informed consent shall then be obtained as soon as permitted based on safety regulations to prevent the transmission of SARS-CoV-2.

The process for obtaining and documenting initial and continued informed consent from potential Trial participants will be conducted in accordance with ICH-GCP E6(R2), applicable regulatory requirements and internal Standard Operating Procedures (SOPs).

All originally signed obtained Informed Consent Forms (ICFs) must be retained/archived in the Investigator Site File (ISF) at the Participating Site and must not be destroyed (even when a scanned copy is available) before expiration of the legal archiving term as defined in the protocol section entitled "Archiving".

Participants may voluntarily withdraw consent to participate in the Trial for any reason at any time. The participant's request to withdraw from the Trial must always be respected without prejudice or consequence to further treatment. Consent withdrawal will be documented in the participant's medical record. The PI must take into account the consequences of such withdrawal: (1) further use of personal data/Trial data, (2) use of human biological materials already collected, (3) safe transition to alternative treatment options, etc. as applicable

4.2 Selection of Participants / Recruitment

Only adult hospitalized patients who tested positive for SARS-CoV-2 will be included.

4.3 Randomization Procedure

To ensure the integrity of the Trial, a randomization procedure through a computerized system has been established , generated by the data management unit of the clinical trial center leuven.

4.4 Trial Procedures

4.4.1 By visit

Screening:
Patients with documented SARS-CoV-2 who require hospitalization will be screened for eligibility.
Informed consent will be obtained. When written informed consent is not possible due to restrictions to prevent the transmission of SARS-CoV-2, oral informed consent will be documented in the medical files, and completed with written informed consent as soon as the restrictions do no longer apply.
Demographic parameters will be obtained. Medical history will be obtained as part or routine clinical care. When study-related procedures impose an additional burden on the clinical care of patients, they can be waived.

Baseline:
Parameters should be obtained as part of routine clinical care.

When study related procedures impose an additional burden on the clinical care of patients, they can be waived. Study drug will be administered when randomized to the investigational drug arm. Medication will be reviewed using the electronic medical files. Serious adverse events and adverse events grade IV will be collected when these are not outcomes of the study.

Daily assessments until discharge:

- Administration of study drug
- Vital signs including SpO2
- Clinical data collection for assessment of study outcomes
- Targeted medication review
- Adverse event evaluation

Serious adverse events and adverse events grade IV will be collected when these are not outcomes of the study. When study-related procedures impose an additional burden on the clinical care of patients, they can be waived.

Visit at Day 15 (+/-2) and 29(+/-5)
These visits can be phone visits when patients are no longer hospitalized.

4.4.2 Laboratory tests

All laboratory tests are part or routine clinical care, but when available will be collected (CRP, white cell count, haemoglobin, platelets, creatinine, hsTroponinT, glucose, total bilirubin, ALT, and AST on days 1; 3, 5, 8, 11, 15 and 29).

4.4.3 Other investigations

The study includes two optional samples on Day 6 (+- 2). on the condition that this does not hinder routine clinical care.

- An additional assessment (e.g. nasopharyngeal swab) for SARS-CoV-2 quantitave PCR
- Plasma sample (Heparine tube) for itraconazole through levels

These samples will be stored and analyzed at a later time point if feasible.

4.5 Premature discontinuation of Trial treatment

Participants may voluntarily discontinue Trial treatment and/or prematurely end their participation in the Trial for any reason at any time. In such case the Investigator must make a reasonable effort to contact the participant (e.g. via telephone, e-mail, letter) in order to document the primary reason for this decision.

The Investigator may also decide at any time during the course of the Trial, to temporarily interrupt or permanently discontinue the Trial treatment if it is deemed that continuation would be detrimental to, or not in the best interest of the participant.

Similarly, the Sponsor, Ethics Committee or authorized regulatory authority can decide to halt or prematurely terminate the Trial when new information becomes available whereby the rights, safety and well-being of Trial participants can no longer be assured, when the integrity of the Trial has been compromised, or when the scientific value of the Trial has become obsolete and/or unjustifiable.

Circumstances requiring premature treatment interruption or discontinuation of the Trial, include but are not limited to:

- Safety concerns related to IMP or unacceptable intolerability
- Trial participation while in violation of the inclusion and/or exclusion criteria

- Pregnancy
- Intention of becoming pregnant
- …

In any such case of early Trial termination and/or treatment interruption/discontinuation, the Investigator will continue to closely monitor the participant's condition and ensure adequate medical care and follow-up.

For participants whose status is unclear because they fail to appear for Trial visits without stating an intention to discontinue or withdraw, the Investigator must make every effort to demonstrate "due diligence" by documenting in the source documents which steps have been taken to contact the participant to clarify their willingness and ability to continue their participation in the Trial (e.g. dates of telephone calls, registered letters, etc.).

A participant should not be considered lost to follow-up until due diligence has been completed.

5 Trial Medication / Drug

**Generic Drug Name** **(& company brand name)**	**IMP or non-IMP**	**Used within Indication? (Y or N)**
Itraconazole (solution 50 mg/5 mL – 150 mL)	IMP	N
Itraconazole capsules 100 mg	IMP	N

5.1 Investigational Medicinal Product and Dosing Regimen

The study design is adaptive, to allow the adjustment of a treatment arm, the addition of a new treatment arms or the removal of a treatment arm based on the most updated information in a rapidly evolving field, based on the continuous assessment of the existing evidence available for the IMP and other potential drug candidates.

Initially, the trial will contain two arms.

Itraconazole is the first example of an active product, but, as noted above, other candidate therapeutics may be considered. Subjects will be randomized to receive either standard of care or standard of care in combination with itraconazole. On day 1-3, itraconazole 200 mg will be administered, as solution or as capsules, three times a day. Subsequently, itraconazole 200 mg twice daily will be administered as an oral solution (50 mg/5mL – 150 mL) or capsule (100 mg) for at least 10 days, and up to Day 15 or discharge.

The capsules (2x 100 mg/gift) will be given to patients without nasogastric tube who are able to consume a meal. Itraconazole capsules will be given in these patients with a meal and with an acidic beverage (non-diet Coca-cola).Concomitant treatment with antacids or acid-suppressive therapy (e.g. protonpump-inhibitors) should be discontinued, if possible.

The oral solution (20 mL/gift) will be administered to patients with a nasogastric tube (and/or enteral feeding). The enteral feeding will be stopped for at least 2h before the administration until at least 1h after the administration. Before and after administration of the oral solution, the tube will be rinsed with 20 mL of water.

The study will randomize participants 1:1 to standard of care or standard of care in combination with the investigational product. If additional arms are added to or dropped from the trial, randomization will proceed with an equal probability of assignment to each of the remaining arms, or a second randomization will be carried out, after review of potential drug interactions. As new interventions are added, the protocol will be amended and reviewed by IRBs/IECs and applicable regulatory agencies before implementation. Drug-drug interactions should be checked thoroughly as itraconazole is a potent CYP3A4 and P-GP inhibitor and also a CYP3A4 substrate itself. Drug Accountability
Investigational drug will be administered to hospitalized patients. The hospital electronic medical prescription will be monitored to assess drug accountability.

5.2 Concomitant / Prohibited Medication / Treatment

There are currently no approved treatments for COVID-19. Patients will receive the standard of care as continuously updated by national and international guidance.

6 Safety

6.1 Specification, timing and recording of safety parameters

- Grade 4 adverse events (life-threathening or urgent intervenstion required)
- SAEs.
- Lab values: CRP, white cell count, haemoglobin, hsTroponinT, platelets, creatinine, glucose, total bilirubin, ALT, and AST on days 1; 3, 5, 8, 11, 15 and 29 (If measured according to clinical indication).
- The electronic medical prescription will be monitored for drug-drug interactions and QT-prolongation.
  Indeed, itraconazole is a potent CYP-3A4 inhibitor and the drug may trigger QT-prolongation. Follow-up of absolute QTc and deltaQTc interval between days 0-2, days 3-5, days 6-8, and days 9-10 of treatment on units with intermittent ECG monitoring or continuous follow-up on ICUs. When QTc> 500 ms and/or delta QTc>60ms, IMP will be interrupted/discontinued at the discretion of the investigator.

6.2 Specification, timing and recording of safety parameters

6.2.1 Definitions

Adverse Event (AE)

An AE is any untoward medical occurrence in a patient or subject during an experiment, and which does not necessarily have a causal relationship with this treatment.

An AE can therefore be any unfavourable and unintended sign (including an abnormal laboratory finding), symptom or disease temporally associated with the use of a product, whether or not considered related to the product. Any worsening (i.e., any clinically significant adverse change in the frequency or intensity of a pre-existing condition) should be considered an AE.

Adverse Reaction (AR) or Adverse Drug Reaction (ADR)

An AR is any untoward and unintended responses to an investigational medicinal product or to an experiment and, when an investigational product is concerned, related to any dose administered.

Serious Adverse Event (SAE)

An SAE is untoward medical occurrence that results in any of the following:

- Death
- A life-threatening[a] experience
- In-patient hospitalisation or prolongation of existing hospitalisation
- A persistent or significant disability or incapacity
- A congenital anomaly or birth defect
- Important medical events that may be considered an SAE when - based on appropriate medical judgement - they may jeopardise the subject and may require medical or surgical intervention to prevent one of the above outcomes

[a] The term "life threatening" in the definition of SAE refers to an event in which the subject was at risk of death at the time of the event. It does not refer to an event which hypothetically might have caused death if it was more severe.

Suspected Unexpected Serious Adverse Reaction (SUSAR)

A SUSAR is an adverse reaction, the nature or severity of which is not consistent with the information on the experiment, and, when a clinical trial is concerned, with the applicable product information (e.g. investigator's brochure for an unauthorised investigational product or the patient leaflet joined to the summary of product characteristics for an authorised product).

6.2.2 Adverse Events that do not require reporting

In general, the following should <u>not be reported as AEs:</u>

- Pre-existing conditions, including those found as a result of screening (these should be reported as medical history or concomitant illness).

- Pre-planned procedures, unless the condition for which the procedure was planned has worsened from the first trial-related activity after the subject has signed the informed consent.

The following events not to be considered as SAEs are:

- Pre-planned hospitalisations unless the condition for which the hospitalisation was planned has worsened from the first trial-related activity after the subject has signed the informed consent.
- Hospitalisation as part of a standard procedure for protocol therapy administration. However, hospitalisation or prolonged hospitalisation for a complication of therapy administration will be reported as an SAE.
- Hospitalisation or prolongation of hospitalisation for technical, practical, or social reasons, in absence of an AE.

For this trial, only Adverse Events graded as severe shall be collected, i.e. adverse events that are life-threatening and/or require an urgent intervention. Adverse events that are also outcomes of the trial, are also exempt from reporting.

6.2.3 Recording and reporting of Adverse Events

Investigators will seek information on AEs during each patient contact. All events, whether reported by the patient or noted by trial staff, will be recorded in the patient's medical record and in the (e)CRF within a reasonable time after becoming aware. If available, the diagnosis should be reported on the AE form, rather than the individual signs or symptoms. If no diagnosis is available, the Investigator should record each sign and symptom as individual AEs.

The following minimum information should be recorded for each AE:

- AE description
- start and stop date of the AE
- severity
- seriousness
- causality assessment to the Investigational Medicinal Product (IMP) and/or study procedures
- outcome

6.2.4 Assessment

All AEs must be evaluated by an Investigator as to:

- **Seriousness:** whether the AE is an SAE. See above for the seriousness criteria.
- **Severity:**
  - Severity must be evaluated by an Investigator according to the following definitions:
    - *Mild* – no or transient symptoms, no interference with the subject's daily activities
    - *Moderate* – marked symptoms, moderate interference with the subject's daily activities
    - *Severe* – considerable interference with the subject's daily activities, unacceptable
- **Causality:**
  - *None* – An AE which is not related to the IMP or experiment
  - *Unlikely* – An AE for which an alternative explanation is more likely (e.g. concomitant medication(s), concomitant disease(s)), and/or the relationship in time suggests that a causal relationship is unlikely
  - *Possible* – An AE which might be due to the use of the IMP or the experiment. An alternative explanation is inconclusive. The relationship in time is reasonable; therefore the causal relationship cannot be ruled out.
  - *Probable* - An AE which might be due to the use of the IMP or the experiment. The relationship in time is suggestive (e.g. confirmed by dechallenge). An alternative explanation is less likely.

- *Definitely* – An AE which is listed as a possible adverse reaction and cannot be reasonably explained by an alternative explanation. The relationship in time is very suggestive (e.g. it is confirmed by dechallenge and rechallenge).

6.2.5 Timelines for reporting

For this trial, only Adverse Events grade 4 shall be collected, i.e. adverse event that are life-threatening and/or require an urgent intervention. Adverse events that are also collected as outcomes will not be separately reported.

All SAEs must be reported to the Sponsor within 24 hours of the trial staff becoming aware of the event. The immediate report shall be followed by detailed, written reports. The immediate and follow-up reports shall identify subjects by Trial identification.

SAE details will be reported by the Investigator to the Sponsor:

- By completing the SAE form in the (e)CRF

6.2.6 Follow-up

The Investigator must record follow-up information by updating the patient's medical records and the appropriate forms in the (e)CRF. The worst case severity and seriousness of an event must be kept throughout the trial.

SAE follow-up information should only include new (e.g. corrections or additional) information and must be reported within 24 hours of the Investigator's first knowledge of the information. This is also the case for previously non-serious AEs which subsequently become SAEs.

- All *SAEs* must be followed up until the outcome of the event is 'recovered', 'recovered with sequelae', 'not recovered' (in case of death due to another cause) or 'death' (due to the SAE) and until all related queries have been resolved, or until end of trial (whichever occurs first).
- *Non-serious AEs* must be followed up until the patient's last study visit, and until all related queries have been resolved.

SAEs after the end of the trial: If the Investigator becomes aware of an SAE with suspected causal relationship to the IMP or experiment after the subject has ended the trial, the Investigator should report this SAE within the same timelines as for SAEs during the trial.

6.2.7 Death

All deaths will be reported without delay to the sponsor (irrespective of whether the death is related to disease progression, the IMP, study procedure or is an unrelated event). The sponsor will notify all deaths, as soon as possible after becoming aware, to the Central EC and the EC of the concerned site and provide additional information if requested.

6.2.8 Reporting requirements to Ethics Committee's (EC's) and Competent Authorities (CA's)

The Investigator is responsible for ensuring that all safety events are recorded in the (e)CRF and reported to the Sponsor in accordance with instructions provided below.

The Sponsor will promptly evaluate all SAEs against medical experience to identify and expeditiously communicate possible new safety findings to Investigators, EC's and applicable CA's based on applicable legislation.

6.2.9 Sponsor's reporting of Suspected Unexpected Serious Adverse Reactions (=SUSARs)

After receiving the SAE report form from the Investigator, the Sponsor has to make a causality (relationship) assessment. The term SADR (Serious Adverse Drug Reaction) is to be used whenever either the Investigator or the Sponsor deems the SAE as possibly or probably related to the IMP.

The Sponsor must evaluate (and document the evaluation of) the expectedness for each SADR against the Reference Safety Information, e.g. in the Investigator's Brochure or applicable product information. In case the event is Unexpected (= a SUSAR) it must be reported by the Sponsor to the EC's, CA's (through the EudraVigilance database) and other participating Investigators within the following timelines:

- **7** calendar days if fatal or life-threatening event (follow-up information within an additional 8 days)
- **15** calendar days if non-fatal or non-life-threatening event (follow-up information as soon as possible)

For reporting to the EudraVigilance database, all information related to the SUSAR should be provided by the Sponsor to the CTC of UZ Leuven as soon as possible. Contact details: CTC@uzleuven.be and tel. 016 34 19 98.

6.2.10 Annual reporting

The Sponsor has the obligation to, once a year throughout the clinical trial (or on request), submit a progress report to the EC's and CA's containing an overview of all SARs occurred during the reporting period and taking into account all new available safety information received during the reporting period.

6.2.11 Data monitoring committee (DMC) and Treatment stopping rules

Given the severity of illness in COVID-19, there are no pre-specified study stopping rules for safety. The protocol team will review AE / SAE data on an ongoing basis. If there are a concerning number of unexpected AEs, the DMC will be asked to review safety data in an ad hoc meeting.

The DMC will review safety data after every 50 subjects are entered into the trial and ad hoc reviews will be undertaken if there are other specific safety concerns. The study will not stop enrolment awaiting these DMC reviews, though the DMC may recommend temporary or permanent cessation of enrolment based on their safety reviews. There are no pre-specified treatment stopping rules.

There interim monitoring will allow early stopping for reasons of futility, inefficacy, or safety. If new effective therapies are identified through these trials, these should become standard of care immediately, in an attempt to control the COVID-19 pandemic as quickly as possible.

7 Statistics and Data Analysis

Statistical analysis will be performed in accordance with ICH E9; a detailed description of the analysis is provided in the Trial-specific Statistical Analysis Plan (SAP). ICH E3 and E8 will guide the structure and content of the clinical trial report. A brief summary is provided here. Details will be described in the SAP.

General considerations:

Adaptive design and blinded interim analysis

This study is intended to allow for two types of adaptations:

1) blinded confirmation or modification of the day selected for the primary outcome analysis and

2) ability to add a new experimental arm if one becomes available.

Blinded endpoint confirmation or modification

The current plan is to evaluate the primary outcome on Day 15, in line with the WHO master protocol. Because there is uncertainty about the clinical course and potential different trajectories according to baseline disease severity, the day of the primary outcome may be modified based on a blinded evaluation of various timepoints (e.g., days 7-21) [6]. This will occur after approximately 100 participants have been enrolled, by a blinded endpoint evaluation committee without knowledge of treatment assignment. Analyses will be evaluated by baseline severity (mild/moderate vs severe). For example, in mild disease, recovery may occur rapidly such that all with mild disease have resumed normal activities by Day 15. Hence, the final timepoint selected may vary accordingly.

Addition of new experimental therapies

If additional data become available to add an experimental therapy, analyses of experimental arms will be performed comparing concurrently enrolled control subjects. If one treatment crosses an efficacy stopping boundary, this treatment may become the new control arm for comparisons. This approach was used in the recent PALM Ebola therapy RCT [7].

Primary outcome

The primary outcome is based on an ordinal severity scale with 7 categories. This scale has been proposed by the WHO for COVID-19 related research and has been previously used in trials of patients with influenza. Previously reported studies and ongoing studies record the same primary outcome, which allows cross-study data pooling.

For the primary analysis, we use a cumulative clinical severity score, based on the 7-category ordinal scale, for the first 15 days (or other time point, based on the blinded interim analysis as described above).

This score is calculated by adding the daily severity score (highest score for that day) for each day from day 1 to day 15, thus providing a cumulative measure of disease severity during the course of the disease.

The null hypothesis being tested is that the mean cumulative clinical severity score during the first 15 days is the same for the standard of care and experimental treatment arms.

7.1 Sample Size Determination

Despite rapid dissemination of data from clinical case series and some early stage clinical trials, detailed information about the course of the disease is limited in this stage of the COVID-19 pandemia. The samples sizes presented here are only illustrative. The larger the number randomised the more accurate the results will be, but the numbers that can be randomised will critically depend on how large the epidemic becomes.

Furthermore, in the absence of treatments with a known benefit, rapid changes in standard of care are to be expected and important signs of a benefit or a harm of a treatment under investigations will require rapid reporting. The interim trial results will be monitored by a Data Monitoring Committee, and if at any stage evidence emerges that any one treatment arm is definitely inferior then it can be decided that that arm will be discontinued. Conversely, if good evidence emerges while the trial is continuing that some other treatment(s) should also be being evaluated then it can be decided that one or more extra arms will be added while the trial is in progress.

Sample size estimates provided as a reference but not to indicate final number of patients to be randomised. Based on the clinical scores on day 7 and day 15 from recently published data [8], we estimate a mean cumulative clinical severity score up to day 15 of 60, with a standard deviation of 20.

With a power of 0.8 and an alpha of 0.05, sample size estimates to detect an 8-point difference in cumulative clinical severity score would require 196 patients (2 times 98), and 502 patients (251 in each group) would be required to detect a 5-point difference with a power of 0.8. We propose an initial sample size of 100 patients per group.

7.2 Statistical Analysis

7.2.1 Population for analysis

The primary analysis will be based on an intention-to-treat population, including all participants randomized. Similarly, safety analyses will be based a modified intent-to- treat population consisting of all participants who received at least one administration of the IMP.

7.2.2 Statistical Analyses

7.2.2.1 General Approach

This is an open label controlled randomized trial testing a superiority hypothesis with a two-sided type I error rate of 0.05. In this explorarory study, secondary hypotheses will be tested in a non-hierachical way. These will be described according to the appropriate summary statistics (e.g., proportions for categorical data, means with 95% confidence intervals for continuous data, median for time-to-event data).

A statistical analysis plan (SAP) will be developed and filed with the study sponsor prior to database lock.

7.2.2.2 Analysis of the Primary Efficacy Endpoint

For the primary analysis, we use a cumulative clinical severity score, based on the 7-category ordinal scale, for the first 15 days (or other time point, based on the blinded interim analysis as described above).

This score is calculated by adding the daily severity score (highest score for that day) for each day from day 1 to day 15, thus providing a cumulative measure of disease severity during the course of the disease.

The null hypothesis being tested is that the mean cumulative clinical severity score during the first 15 days is the same for the standard of care and experimental treatment arms. Because means of summed scores over a number of days are expected to be symmetrically distributed, we will use a t-test to compare the mean cumulative clinical severity score on day 15 between the treatment and the standard of care group.

As a co-primary endpoint, according to the WHO suggestions, we will use the ordinal scale to estimate a proportional odds model. For this model, the primary hypothesis test will be based on a test of whether the common odds ratio for treatment is equal to one. For large sample sizes, the hypothesis test is nearly the same as the Wilcoxon rank sum test.

Therefore, the procedure produces a valid p-value regardless of whether the proportional odds model is correct. Nonetheless, estimation and confidence intervals do require the model to be correct. Accordingly, we will evaluate model fit using a goodness-of-fit likelihood ratio test. A stratified hypothesis test to account for baseline severity of disease will be used.

7.2.2.3 Analysis of the Secondary Endpoint(s)

1. Differences in time-to-event endpoints (e.g., time to a one category improvement in ordinal scale) by treatment will be summarized with Kaplan- Meier curves and 95% confidence bounds.
2. Change in ordinal scale at specific time points will be summarized by proportions (e.g., proportion who have a 1-, 2-, 3-, or 4-point improvement or 1-, 2-, 3-, 4-point worsening).
3. Duration of event (e.g., duration of mechanical ventilation) will be summarized according to median days with quartiles.
4. Incidence data (e.g., incidence of new oxygen use) will be summarized as a percent with 95% confidence intervals.
5. Categorical data (e.g., 28-day mortality or ordinal scale by day) will be summarized according to proportions with confidence intervals on the difference or odds ratios for a binary or multiple category scale, respectively.

Missing data procedures will be described in the SAP.

7.2.2.4 Safety Analyses

Safety endpoints are described above. These events will be analysed univariately and as a composite endpoint. Time-to-event methods will be used for death and the composite endpoint. Each AE will be counted once for a given participant and graded by severity and relationship to COVID-19 or study intervention.

Adverse events leading to premature discontinuation from the study intervention and serious treatment-emergent AEs will be described as part of the primary publication of the study results.

7.2.2.5 Baseline Descriptive Statistics

Baseline characteristics will be summarized by treatment arm. For continuous measures the mean and standard deviation will be summarized. Categorical variables will be described by the proportion in each category (with the corresponding sample size numbers).

7.2.2.6 Planned Interim and Early Analyses

Early analysis

An initial blinded endpoint-evaluation phase will be enrolled prior to specification of the primary endpoint as described above. Analysis and decision making will be restricted to a blinded endpoint evaluation committee (a BEEC). BEEC membership will be defined elsewhere and will consist only of individuals who are blinded to treatment assignment. Principles of blinded endpoint-evaluation will be defined in a separate document.

Additional early analyses include monitoring enrolment, baseline characteristics, and follow-up rates throughout the course of the study by the study team. Analyses will be conducted blinded to treatment assignment.

Interim analyses

An independent data monitoring committee (iDMC) will monitor ongoing results to ensure patient well-being and safety as well as study integrity. The iDMCwill be asked to recommend early termination or modification only when there is clear and substantial evidence of a treatment difference.

7.2.2.7 Sub-Group Analyses

Subgroup analyses for the primary and selected secondary outcomes will evaluate the treatment effect across the following subgroups: duration of symptoms prior to enrolment, age groups, disease severity at baseline and co-morbidities. A forest plot will display confidence intervals across subgroups. Interaction tests will be conducted to determine whether the effect of treatment varies by subgroup.

7.3 Trial Steering Committee (TSC) / Data Monitoring Committee (iDMC)

The iDMC will review safety data after every 50 subjects are entered into the trial and ad hoc reviews will be undertaken if there are other specific safety concerns. The study will not stop enrolment awaiting these iDMC reviews, though the iDMC may recommend temporary or permanent cessation of enrolment based on their safety reviews. There are no pre-specified treatment stopping rules.
Given the severity of illness in COVID-19, there are no pre-specified study stopping rules for safety. The protocol team will review AE / SAE data on an ongoing basis. If there are a concerning number of unexpected AEs, the iDMC will be asked to review safety data in an ad hoc meeting.

There interim monitoring will allow to recommend early stopping for reasons of futility, inefficacy, or safety. If new effective therapies are identified through these trials, these snould become standard of care immediately, in an attempt to control the COVID-19 pandemic as quickly as possible.

8 Data handling

8.1 Data Collection Tools and Source Document Identification

Data collection, handling, processing and transfer for the purpose of this Trial will be performed in compliance with applicable regulations, guidelines for clinical trials and internal procedures, as follows:

8.1.1.1 Data collection

Source Data will be collected and recorded in the Trial participant's files/medical records.

Worksheets may be used for capturing some specific data in order to facilitate completion of the eCRF. Any such worksheets will become part of the Trial participant's source documentation and will be filed together with or as part of the medical records (during but also following completion of the Trial).

It remains the responsibility of the Investigator to check that all data relating to the Trial, as specified in the Trial protocol, are entered into the eCRF in accordance with the instructions provided and that the forms are filled out accurately, completely and in a timely manner.

eCRFs are provided by the Sponsor for each participant. The Trial data will be transcribed from the source records (i.e. participant's medical file or Trial-specific source data worksheets) into an eCRF by Trial Staff. Transcription to the eCRF will be done as soon as possible after a participant visit and in a pseudonymized manner using a unique identifier assigned by the Sponsor.

The eCRFs will be available for review at the next scheduled monitoring visit (as applicable).

8.1.1.2 Data Validation

All data relating to the Trial must be prepared and validated by the Investigator. Any eCRF entries, corrections and alterations must be made by the Investigator or other authorized Trial staff.

Proper audit trails are available in REDCap to demonstrate the validity of the Trial data collected. This includes historical records of original data entries, by whom and when the data was entered, as well as detailed records of any corrections or additions made to the original data entry (i.e. who made the correction/addition, when and why), without obliterating the original data entry information.

8.1.1.3 Data Management

The Trial Data Manager will perform extensive consistency checks on the received data. Queries will be issued in case of inconsistencies in accordance with internal procedures. A Data Management Plan will be developed to map data flows, data validation measures that will be taken, how (interim) database lock(s) will be managed and, as applicable, the role and responsibilities of the Data Safety Monitoring Committee (DSMB)

8.1.1.4 Data Transfer

Any participant records or datasets that are transferred to the Sponsor or any partners of the Sponsor will contain the Trial-specific participant identifier only; participant names or any information which would make the participant identifiable will not be transferred. All pseudonymized data relating to the Trial must be transmitted in a secure manner to the Sponsor (see 8.1.2. legal requirements).

8.1.2 Legal requirements

All source data will be kept at a secured location with restricted access at all times. These data must be collected and processed with adequate precautions to ensure confidentiality and compliance with applicable data protection laws and regulations and more in particular the EU General Data Protection Regulation 2016/679 (GDPR) and relevant national laws implementing the GDPR. Appropriate technical and organizational measures to protect the data against unauthorized disclosure or access, accidental or unlawful destruction, or accidental loss or alteration must be established. Trial staff whose responsibilities require access to personal data agree to keep the data confidential.

The Investigator and the Participating Site(s) (as applicable) shall treat all information and data relating to the Trial disclosed to them as confidential and shall not disclose such information to any third parties or

use such information for any purpose other than the objectives of the Trial as described in this protocol. The collection, processing and disclosure of personal data, such as participant health and medical information is subject to compliance with applicable laws and regulations regarding personal data protection and the processing of personal data.

The Investigator will maintain all source documents and completed eCRF that support the data collected from each Trial participant, and will maintain a Trial Master File (TMF) containing all Trial documents as specified in ICH-GCP E6(R2) Chapter 8 entitled "Essential Documents for the Conduct of a Clinical Trial", and as specified by applicable regulatory requirement(s).
The Investigator will take appropriate measures to prevent accidental or premature destruction of these documents.

Transfer of the pseudonymized data will be performed via a secured method of transfer taking into account all applicable security arrangements and regulations (such as the European General Data Protection Regulation). The receiving party will be bound by contractual agreement to keep the transferred data confidential at all times and to only process the data for the purpose of the Trial. To this end, appropriate Data Transfer Agreements (DTAs) will be established.

8.2 Audits and Inspections

The Investigator will permit direct access to Trial data and documents for the purpose of monitoring, audits and/or inspections by authorized entities such as but not limited to: the Sponsor or its designees and competent regulatory or health authorities. As such eCRFs, source records and other Trial related documentation (e.g. the Trial Master File, pharmacy records, etc.) must be kept current, complete and accurate at all times.

8.3 Monitoring

In accordance with ICH-GCP E6(R2) the Sponsor is responsible for monitoring the Trial to ensure compliance with GCP and current legislation, and to verify, among other requirements, that proper written informed consent has been obtained and documented, that the Trial procedures have been followed as shown in the approved protocol, and that relevant Trial data have been collected and reported in a manner that assures data integrity. To this end Source Data will be compared with the data recorded in the eCRF. Monitoring of the Trial will be performed by qualified individuals (independent from the site Trial staff) according to the monitoring plan. The Sponsor and Investigator/Participating Site will permit direct access to the Trial data and corresponding Source Data and to any other Trial related documents or materials to verify the accuracy and completeness of the data collected. More details about the monitoring strategy are described in the Trial specific Monitoring Plan (MP).

8.4 Archiving

As specified in ICH-GCP E6(R2) section 8.1 Addendum the Sponsor and Investigator/Participating Site will maintain a record of the location(s) of all respective Essential Trial Documents (including but not limited to Source Documents, completed and final eCRF and ISF(s)/TMF). The Sponsor should ensure that the Investigator has control of and continuous access to the eCRF data reported to the Sponsor during the Trial.

The Investigator/Participating Site should have control of all Essential Documents and records generated by the Investigator/Participating Site before, during and following termination of the Trial.

The Sponsor is responsible for archiving Trial specific documentation (such as but not limited to the Trial protocol, any amendments thereto, the final Clinical Study Report (CSR) and the Trial database) according to ICH-GCP E6(R2). Source data and site-specific Trial documents (such as but not limited to the original signed ICFs) will be archived by the participating site(s) according to local practice, and for at least 25 years following termination of the Trial. Archived data may be held on electronic record, provided that media back-up exists, hard copies can be obtained, if required and measures are taken to prevent accidental or premature loss or destruction of data. Destruction of Essential Documents will require written authorisation from the Sponsor.

9 Ethical and Regulatory Considerations

9.1 Ethics Committee (EC) review & reports

Before the start of the Trial, this protocol and other related documents (e.g. ICF, advertisements, IB, etc.) will be submitted for review to the EC and to the relevant CA for Trial authorization. The Trial shall not commence until such approvals have been obtained.

It is the responsibility of the CI to produce the Annual Progress Report (APR) and submit to the EC/CA within 30 days of the anniversary date on which favourable opinion to start the Trial was given, and annually until the Trial is declared ended.

The CI shall notify the EC/CA of the end of the Trial. Should the Trial be ended prematurely, the CI will notify the EC/CA and include the reasons for premature termination within 15 days of the decision. The CI will submit a final report with the results, including any publications/abstracts, to the EC/CA within 1 year or within 6 months for paediatric Trials.

9.2 Regulatory Compliance

The Trial will be conducted in compliance with the principles outlined in the requirements for the conduct of clinical Trials in the EU as provided for in Directive 2001/20/EC or EU Regulation 536/2014, as applicable, and any subsequent amendments, as well as in compliance with ICH-GCP E6(R2) guidelines, other GxP guidelines, the most recent version of the Declaration of Helsinki, the Belgian law of May 7th 2004 regarding experiments on the human person (as amended) or the Belgian law of May 7th 2017 on clinical Trials with medicinal products for human use, as applicable, and with the EU General Data Protection Regulation 2016/679 (GDPR), the relevant Belgian laws implementing the GDPR, the Belgian Law of August 22nd 2002 on patient rights and all other applicable legal and regulatory requirements.

9.3 Protocol / GCP compliance

The Trial must be performed in accordance with the protocol, current ICH-GCP guidelines, and applicable regulatory and country-specific requirements. GCP is an international ethical and scientific quality standard for designing, conducting, recording, and reporting studies that involve the participation of human participants. Compliance with this standard provides public assurance that the rights, safety, and well-being of Trial participants are protected, consistent with the principles that originated in the most recent version of the Declaration of Helsinki, and that the Trial data are credible, reliable and reproducible.

The Investigator and Trial team acknowledge and agree that prospective, planned deviations or waivers to the protocol are not permitted under applicable regulations on clinical studies. However, should there be an accidental protocol deviation, such deviation shall be adequately documented in the source documents and on the relevant forms and reported to the CI and Sponsor. Deviations should also be reported to the EC as part of the EC's continued review of the Trial (e.g. through the ASR, APR, etc.). Protocol deviations which are found to frequently recur, will require (immediate) action. Investigator acknowledges that such recurring protocol deviations could potentially be classified as a serious violation.

It is understood that "a serious violation" is likely to affect to a significant degree:

- the safety or physical or mental integrity of the Trial participants; or
- the scientific validity of the Trial

The Investigator is expected to take any immediate action required to protect the safety of any participant included in the Trial, even if this action represents a deviation from the protocol. In such cases, the Sponsor should be notified of this action and the EC at the Trial site should be informed according to local procedures and regulations.

9.4 Data protection and participant confidentiality

The Trial will be conducted in compliance with the requirements of the EU General Data Protection Regulation 2016/679 (GDPR), the relevant Belgian laws implementing the GDPR including the Belgian Privacy Act of 30 July 2018 on the protection of privacy in relation to the processing of personal data. Any collection, processing and disclosure of personal data, such as participant health and medical information is subject to compliance with the aforementioned personal data protection laws (cfr. Data

Processing Annex (DPA) in Appendix). In case personal data is transferred outside the European Economic Area, safeguards will be taken to ensure that appropriate protection travels with the data in accordance with the GDPR. (https://ec.europa.eu/info/law/law-topic/data-protection/international-dimension-data-protection/rules-international-data-transfers_en#documents)

Any personal data shall be treated as confidential at all times including during collection, handling and use or processing, and the personal data (including in any electronic format) shall be stored securely at all times and with all technical and organizational security measures that would be necessary for compliance with EU and national data protection legislation (whichever is more stringent). The Sponsor shall take appropriate measures to ensure the security of all personal data and guard against unauthorized access thereto or disclosure thereof or loss or destruction while in its custody.

9.5 Insurance

The Participating Site, the Investigator and Sponsor shall have and maintain in full force and effect during the term of this Trial, and for a reasonable period following termination of the Trial, adequate insurance coverage for: (i) medical professional and/or medical malpractice liability, and (ii) general liability.

For Belgian Participating Sites

Art 29 of the Belgian Law relating to experiments on human persons dated May 7th, 2004 applies.
Prior to the start of the Trial, the Sponsor shall enter into an insurance contract in order to adequately cover Trial participants from Belgian sites in accordance with art. 29 of the said law.

For non-Belgian Participating Sites

The Participating Site shall have and maintain in full force and effect during the term of this Trial (and for a reasonable period following termination of the Trial, adequate insurance coverage for other possible damages resulting from the Trial at the Participating Site, as required by local law. Each such insurance coverage shall be in amounts appropriate to the conduct of the services of the Participating Site under this Trial. The Participating Site and Sponsor shall be solely responsible for any deductible or self-insured retention under any such policies.

9.6 Amendments

Unless for urgent reasons as specified in ICH-GCP E6(R2) section 4.5.4, amendments must not be implemented prior to EC and/or CA review and/or approval, as applicable.
In accordance with the Belgian law of May 7th 2004 regarding experiments on humans, the Sponsor may develop a non-substantial amendment at any time during the Trial. If a substantial amendment to the clinical Trial agreement or the documents that supported the original application for the clinical Trial authorisation is needed, the Sponsor must submit a valid substantial amendment to the Competent Authority (CA) for consideration, and to the EC for review and approval. The CA and/or EC will provide a response in accordance with timelines defined by applicable regulations. It is the Sponsor's responsibility to assess whether an amendment is substantial or non-substantial for the purpose of submission to the CA and/or EC.

Amendments to the Trial are regarded as 'substantial' when they are likely to have a significant impact on the safety or physical or mental integrity of the clinical Trial participants, or the scientific value of the Trial.

https://ec.europa.eu/health/sites/health/files/files/eudralex/vol-10/2010_c82_01/2010_c82_01_en.pdf

9.7 Post-Trial activities

Not applicable.

10 Research Registration, Dissemination of Results and Publication Policy

The Declaration of Helsinki (latest version) and European and Belgian regulations require that every research Trial involving human participants be registered in a publicly accessible database before recruitment of the first participant. The CI is responsible for registering the Trial.

In addition, the CI will fulfil their ethical obligation to disseminate and make the research results publicly available. As such the CI is accountable for the timeliness, completeness and accuracy of the reports. Researchers, authors, Sponsors, editors and publishers must adhere to accepted guidelines for ethical reporting. Negative and inconclusive, as well as positive results must be published or otherwise made publicly available. Sources of funding, institutional affiliations and conflicts of interest must be declared in publication.

Publications will be coordinated by the CI. Authorship to publications will be determined in accordance with the requirements published by the International Committee of Medical Journal Editors and in accordance with the requirements of the respective medical journal.

For multi-centric Trials, it is anticipated that the primary results of the overall Trial shall be published in a multi-centre publication.

Participating Sites are not allowed to publish any subset data or results from the Trial prior to such multicentre publication.

Any publication by a Participating Site must be submitted to the Sponsor for review at least thirty (30) calendar days prior to submission or disclosure. Sponsor shall have the right to delay the projected publication for a period of up to three (3) months from the date of first submission to the Sponsor in order to enable the Sponsor to take steps to protect its intellectual property rights and know-how.

11 Intellectual Property

Any know-how, inventions, methods, developments, innovations, discoveries and therapies, whether patentable or not, arising from the Trial or made in the performance of the Trial protocol ("Inventions") shall vest in the Sponsor. The Participating Site, its employees and Investigator(s) shall promptly disclose to the Sponsor any such Inventions. Parties have expressly agreed that any and all Trial data as collected and prepared in the performance of the Trial protocol shall be the sole property of Sponsor.

12 Joint Commission International (JCI)

In order to ensure the same quality and safety standards in patient care for clinical research as commonly applied by the Sponsor in its regular activities, and in accordance with JCI standards, the Sponsor shall comply with the following obligations: (a) the Sponsor will use trained and qualified employees or contractors to manage and coordinate the Trial; (b) the Sponsor will ensure that multi-center Trial reporting is reliable and valid, statistically accurate, ethical, and unbiased. (c) the Sponsor will not grant incentives, other than standard compensations and reimbursement of costs, to Trial participants or to participating site's staff that would compromise the integrity of the research; (d) the Sponsor is responsible for monitoring and evaluating the quality, safety, and ethics of the Trial and will respect the participating site's policies and processes when performing such monitoring and evaluation activities; (e) the Sponsor will protect the privacy and confidentiality of the Trial participants in accordance with all applicable laws.

References

1 Zhou, F. *et al.* Clinical course and risk factors for mortality of adult inpatients with COVID-19 in Wuhan, China: a retrospective cohort study. *The Lancet*, doi:10.1016/s0140-6736(20)30566-3 (2020).

2 Keyaerts, E., Vijgen, L., Maes, P., Neyts, J. & Van Ranst, M. In vitro inhibition of severe acute respiratory syndrome coronavirus by chloroquine. *Biochem Biophys Res Commun* **323**, 264-268, doi:10.1016/j.bbrc.2004.08.085 (2004).

3 Wu, C. *et al.* Analysis of therapeutic targets for SARS-CoV-2 and discovery of potential drugs by computational methods. *Acta Pharmaceutica Sinica B*, doi:10.1016/j.apsb.2020.02.008 (2020).

4 Takano, T., Akiyama, M., Doki, T. & Hohdatsu, T. Antiviral activity of itraconazole against type I feline coronavirus infection. *Vet Res* **50**, 5, doi:10.1186/s13567-019-0625-3 (2019).

5 Schloer, S. *et al.* The clinically licensed antifungal drug itraconazole inhibits influenza virus in vitro and in vivo. *Emerg Microbes Infect* **8**, 80-93, doi:10.1080/22221751.2018.1559709 (2019).

6 Posch, M. & Proschan, M. A. Unplanned adaptations before breaking the blind. *Stat Med* **31**, 4146-4153, doi:10.1002/sim.5361 (2012).

7 Mulangu, S. *et al.* A Randomized, Controlled Trial of Ebola Virus Disease Therapeutics. *N Engl J Med* **381**, 2293-2303, doi:10.1056/NEJMoa1910993 (2019).

8 Cao, B. *et al.* A Trial of Lopinavir-Ritonavir in Adults Hospitalized with Severe Covid-19. *N Engl J Med*, doi:10.1056/NEJMoa2001282 (2020).

APPENDICES

13 Appendix I: Data Processing Annex (DPA) (in case the study will become multicentric)

Definitions:

- "Protocol" means the document entitled "A randomized, open-label, adaptive, proof-of-concept clinical trial of new antiviral drug candidates against SARS-CoV-2" containing the details of the academic Trial as developed by the Sponsor and approved by the relevant Ethics Committee.
- "Sponsor" means University Hospitals Leuven (UZ Leuven).
- Participating site acts as a data processor as defined under article 4, 8) of the Regulation (EU) 2016/679 ("Data Processor") for the Sponsor who acts as data controller as defined under article 4, 7) of the Regulation (EU) 2016/679 ("Data Controller").
- "Applicable Law" means any applicable data protection or privacy laws, including:
    a) the Regulation (EU) 2016/679 also referred as the General Data Protection Regulation ("GDPR");
    b) other applicable laws that are similar or equivalent to or that are intended to or implement the laws that are identified in (a) of this definition;
- "Personal Data" means any information relating to an identified or identifiable natural person ("Data Participant"), including without limitation pseudonymized information, as defined in Applicable Law and described in the Protocol.

Rights and obligations:

1. The Data Processor is instructed to process the Personal Data for the term of the Trial and only for the purposes of providing the data processing tasks set out in the Protocol. The Data Processor may not process or use Personal Data for any purpose other than a Data Participant's medical records, or other than provided in the instructions of the Trial protocol, including with regard to transfers of personal data to a third country or an international organization, unless the Data Processor is required to do so according to Union or Member State law.
2. Data Processor shall at all times maintain a record of processing of Personal Data in accordance with Applicable Law and if the Data Processor considers an instruction from the Data Controller to be in violation of the Applicable Law, the Data Processor shall promptly inform the Data Controller in writing about this.
3. The Data Processor must ensure that persons authorized to process the Personal Data have committed themselves to confidentiality or are under an appropriate statutory obligation of confidentiality.
4. The Data Processor shall implement appropriate technical and organizational measures to prevent that the Personal Data processed is:
    (i) accidentally or unlawfully destroyed, lost or altered,
    (ii) disclosed or made available without authorization, or
    (iii) otherwise processed in violation of Applicable Law.
5. The appropriate technical and organizational security measures must be determined with due regard for:
    (i) the current state of the art,
    (ii) the cost of their implementation, and

(iii) the nature, scope, context and purposes of processing as well as the risk of varying likelihood and severity for the rights and freedoms of natural persons.

6. Taking into account the nature of the processing, the Data Processor shall assist the Data Controller, by means of appropriate technical and organizational measures, insofar as this is possible, in fulfilling its obligation to respond to requests from Data Participants pursuant to laws and regulations in the area of privacy and data protection (such as, the right of access, the right to rectification, the right to erasure, the right to restrict the processing, the right to data portability and the right to object)

7. The Data Processor shall upon request provide the Data Controller with sufficient information to enable the Data Controller to ensure that the Data Processor's obligations under this DPA are complied with, including ensuring that the appropriate technical and organizational security measures have been implemented.

8. The Data Controller is entitled to appoint at its own cost an independent expert, reasonably acceptable to the Data Processor, who shall have access to the Data Processor's data processing facilities and receive the necessary information for the sole purpose of auditing whether the Data Processor has implemented and maintained said technical and organizational security measures. The expert shall upon the Data Processor's request sign a non-disclosure agreement provided by the Data Processor, and treat all information obtained or received from the Data Processor confidentially, and may only pass on, after conferral with the Data Processor, the findings as described under 10) (ii) below to the Data Controller.

9. The Data Processor must give authorities who by Union or Member State law have a right to enter the Data Controller's or the Data Controller's processors' facilities, or representatives of the authorities, access to the Data Processor's physical facilities against proper proof of identity and mandate, during normal business hours and upon reasonable prior written notice.

10. The Data Processor must without undue delay in writing notify the Data Controller about:

    (i) any request for disclosure of Personal Data processed under the Protocol by authorities, unless expressly prohibited under Union or Member State law,

    (ii) any finding of (a) breach of security that results in accidental or unlawful destruction, loss, alteration, unauthorized disclosure of, or access to, Personal Data transmitted, stored or otherwise processed by the Data Processor under the Protocol, or (b) other failure to comply with the Data Processor's obligations, or

    (iii) any request for access to the Personal Data (with the exception of medical records for which the Data Processor is considered data controller) received directly from the Data Participants or from third parties.

11. Such a notification from the Data Processor to the Data Controller with regard to a breach of security as meant in 10) (ii)(a) above will contain at least the following information:

    (i) the nature of the Personal Data breach, stating the categories and (by approximation) the number of Data Participants concerned, and stating the categories and (by approximation) the number of the personal data registers affected (datasets);

    (ii) the likely consequences of the Personal Data breach;

    (iii) a proposal for measures to be taken to address the Personal Data breach, including (where appropriate) measures to mitigate any possible adverse effects of such breach.

12. The Data Processor shall document (and shall keep such documentation available for the Data Controller) any Personal Data breaches, including the facts related to the Personal Data breach, its effects and the corrective measures taken. After consulting with the Data Controller, the Data Processor shall take any measures needed to limit the (possible) adverse effects of Personal Data breaches (unless such consultation cannot be awaited due to the nature of the Personal Data breach).

13. The Data Processor must promptly and reasonably assist the Data Controller (with the handling of (a) responses to any breach of security as described in 10) (ii) above and (b) any requests from

Data Participants under Chapter III of the GDPR, including requests for access, rectification, blocking or deletion. The Data Processor must also reasonably assist the Data Controller by implementing appropriate technical and organizational measures for the fulfilment of the Data Controller's obligation to respond to such requests.

14. The Data Processor must reasonably assist the Data Controller with meeting the other obligations that may be incumbent on the Data Controller according to Union or Member State law where the assistance of the Data Processor is implied, and where the assistance of the Data Processor is necessary for the Data Controller to comply with its obligations. This includes, but is not limited to, at the request to provide the Data Controller with all necessary information about an incident under 10) (ii), and all necessary information for an impact assessment in accordance with Article 35 and Article 36 of the GDPR.

Subprocessor:

15. The Data Processor may only engage a subprocessor, with prior specific or general written consent from the Data Controller. The Data Processor undertakes to inform the Data Controller of any intended changes concerning the addition or replacement of a subprocessor by providing a reasonable prior written notice to the Data Controller. The Data Controller may reasonably and in a duly substantiated manner object to the use of a subprocessor. The Data Processor must inform the Data Controller in writing of the discontinued use of a subprocessor.

16. Prior to the engagement of a subprocessor, the Data Processor shall conclude a written agreement with the subprocessor, in which at least the same data protection obligations as set out in this DPA shall be imposed on the subprocessor, including obligations to implement appropriate technical and organizational measures and to ensure that the transfer of Personal Data is done in such a manner that the processing will meet the requirements of the Applicable Law.

17. The Data Controller has the right to receive a copy of the relevant provisions of Data Processor's agreement with the subprocessor related to data protection obligations. The Data Processor shall remain fully liable to the Data Controller for the performance of the subprocessor obligations under this DPA. The fact that the Data Controller has given consent to the Data Processor's use of a subprocessor is without prejudice for the Data Processor's duty to comply with this DPA.

14 Appendix 2: Drug-Drug Interactions Itraconazole

Category X: drugs that should not be administered together with itraconazole

**Drug molecule**	**Comments**
alfusozine	
aliskiren	
alprazolam	
aprepitant	
avanafil	

bilastine	Alternatives to bilastine therapy should be considered. Bilastine therapy should be avoided in patients with moderate to severe renal insufficiency receiving itraconazole due to an increased risk of adverse effects of bilastine (e.g. QTc-prolongation).
bosutininb	
bromocriptine	
cobimetinib	
dapoxetine	
disopyramide	
domperidone	
doxorubicin	
eletriptan	
eliglustat	
eplerenone	
ergotamine	
everolimus	
felodipine	
fluticasone	
fosaprepitant	
ibrutinib	Interrupt ibrutinib therapy if short-term treatment with itraconazole is necessary.
idelalisib	
irinotecan	Avoid itraconazole during and within 1 week prior to irinotecan.
isavuconazole	
ivabradine	
lercanidipine	
macitentan	
methadone	

midazolam (oral)	
mizolastine	
nimodipine	
pazopanib	
pimozide	
reboxetin	
red yeast rice	Switch red yeast rice to fluvastatin, rosuvastatin, pravastatin.
regorafenib	
rifampicin	
rivaroxaban	
rupatadine	
Saccharomyces boulardii	
salmeterol	
sildenafil	**Pulmonary arterial hypertension (PAH):** combination with itraconazol is not recommended.
silodosin	
simvastatin	Switch simvastatin to fluvastatin, rosuvastatin, pravastatin.
tadalafil	For treatment of **pulmonary arterial hypertension** in patients who are also taking itraconazole
tamsulosine	
temsirolimus	
ticagrelor	
tolvaptan	
topotecan	
trabectedin	If itraconazole is used for less than 14 days, administer itraconazole 1 week after the trabectedin infusion, and discontinue it the day prior to the next trabectedin infusion.
triazolam	

ulipristal	
vemurafenib	Coadministration of itraconazole and vemurafenib increased vemurafenib AUC by approximately 40%
vincristine	

Category D: drugs that require dose reduction when administered together with itraconazole

Drug molecule	Comments
abemaciclib	In patients taking abemaciclib at a dose of 200 mg twice daily or 150 mg twice daily, reduce the abemaciclib dose to 100 mg twice daily. In patients who have had a dose reduction to 100 mg twice daily due to adverse reactions, further reduce the abemaciclib dose to 50 mg twice daily.
almotriptan	Use an initial almotriptan dose of 6.25mg when using almotriptan with itraconazole, and do not exceed 12.5mg of almotriptan in any 24-hour period. Avoid concurrent use of almotriptan with itraconazole in patients with impaired hepatic or renal function.
apixaban	Per apixaban US prescribing information, patients receiving itraconazole should receive apixaban at 50% of the usual dose if they would otherwise receive 5 mg or 10 mg twice daily, and should not receive apixaban if they would otherwise receive 2.5 mg twice daily. At least some non-US apixaban labeling states that this combination is contraindicated.
aripiprazole (Extended release)	The following dose adjustments are recommended only when inhibitors are given for more than 14 days. In patients who would normally receive a 400 mg aripiprazole dose, reduce dose to 300 mg when given with a strong CYP3A4 inhibitor and further to 200 mg in patients who are also receiving CYP2D6 inhibitors or who are CYP2D6 poor metabolizers; in patients who would normally receive a 300 mg dose, reduce dose to 200 mg when given with a strong CYP3A4 inhibitor and further to 160 mg in patients who are also receiving CYP2D6 inhibitors or who are CYP2D6 poor metabolizers. Increase aripiprazole doses proportionately following discontinuation of treatment with CYP3A4 and/or CYP2D6 inhibitors.
aripiprazole (Immediate release)	Decrease the aripiprazole dose to 50% of the usual dose when initiating concomitant therapy with a strong CYP3A4 inhibitor, and further to 25% of the usual dose in patients who are also receiving strong CYP2D6 inhibitors (e.g., paroxetine, quinidine) or who are CYP2D6 poor metabolizers. Dose reductions up to 75% could also be considered when combining a strong CYP3A4 inhibitor with a less potent CYP2D6 inhibitor.
atorvastatin	Limit atorvastatin to a maximum adult dose of 20 mg/d. Monitor for toxic effects of atorvastatin (e.g., myalgia, rhabdomyolysis, liver function test abnormalities) or switch to alternative HMG-CoA reductase inhibitors: fluvastatin, rosuvastatin,pravastatin.
axitinib	Decrease the axitinib dose by 50%, with any subsequent dose modifications based on patient response. If itraconazole is discontinued, the axitinib dose should be returned (after 3 to 5 half-lives of the inhibitor) to that used prior to initiation of itraconazole.
cabazitaxel	Avoid concurrent use; if not possible, reduce dose Cabazitaxel with 25%.

cabozantinib	Avoid concurrent use; if not possible, reduce cabozantinib dose by 40 mg from the previous dose for medullary tyroid cancer and by 20 mg for renal cell and hepatocellular carcinoma.
cariprazine	When on stable cariprazine dose, reduce dose of cariprazine by half. When already on itraconazole, initiate cariprazine therapy with 1.5mg on d1, no dose on d2, then 1.5mg daily, increasing to a max of 3mg/dag as appropriate.
ceritinib	Avoid concurrent use; if not possible reduce ceritinib dose by 33%
cobicistat	Max. adult dose of itraconazole of 200mg/day when used concurrent with combination products containing Cobicistat. Monitor for Itraconazole and Cobicistat toxicity.
colchicine	Reduce dose dependent on indication and colchicine brand cfr. UpToDate.
crizotinib	Avoid Concurrent use; if not possible, decrease crizotinib dose to 250mg/d. Monitor for crizotinib toxicity (QTc interval, ventricular arrhythmias).
cyclosporine	Reduce cyclosporine dose with 50% to 80% and monitor cyclosporin concentrations closely.
darifenacin	Max. darifenacin dose of 7.5mg/d. Monitor for increased darifenacin toxicities.
darunavir	Max. adult dose of itraconazole of 200mg/day.
dasatinib	Avoid concurrent use; if not possible, reduce dasatinib dose from 140mg to 100mg; from 70-100mg to 20mg depending on starting dose. Stop Dasatinib if starting dose is 40-60mg until discontinuation of itraconazole.
digoxin	Consider dose reduction of Digoxin when Itraconazole is initiated. Monitor for cardiac glycoside toxicity.
docetaxel	Avoid concurrent use; if not possible, reduce docetaxel dose by 50% and monitor for increased docetaxel toxicity.
edoxaban	Indication: venous thromboembolism, limit edoxaban dose to 30 mg daily. No dose adjustment is recommended when indication for edoxaban is treatment of nonvalvular atrial fibrillation.
elvitegravir	Maximum 200mg itraconazole per day.
encorafenib	For patients taking encorafenib 450 mg: Decrease dose to 150 mg. For patients taking encorafenib 300 mg: Decrease dose to 75 mg. For patients taking encorafenib 225 mg: Decrease dose to 75 mg.
everolimus	Dose adjustments may be needed, especially if combined with another P-gp inhibitor or moderate CYP3A4 inhibitor.
fesoterodine	Avoid fesoterodine doses greater than 4mg daily in patients who are also receiving itraconazole. Itraconazole US prescribing information lists its use with fesoterodine as contraindicated in patients with moderate to severe renal or hepatic impairment.

fosamprenavir	Limit the adult maximum itraconazole dose to 200 mg/day in patients receiving fosamprenavir/ritonavir. In patients receiving fosamprenavir without ritonavir, itraconazole dose reductions may be needed for patients receiving itraconazole doses greater than 400 mg/day.
fosnetupitant	Avoid concurrent use; if not possible, a reduced dose of itraconazole may be necessary.
guanfacine	Reduce the guanfacine dose by 50% when initiating concomitant therapy with itraconazole. When discontinuing itraconazole treatment, increase the guanfacine dose to the recommended dose range. Monitor closely for evidence of excessive guanfacine response (eg.hypotension, bradycardia, CNS depression).
ivacaftor	Administer two ivacaftor tablets (75 mg) in the morning, twice a week, approximately 3 to 4 days apart.
lapatinib	Avoid concurrent use; if not possible, reduce lapatinib dose to 500mg/d. Increase dose back to normal 1 week after discontinuation of itraconazole.
lopinavir	Max. adult dose of itraconazole of 200 mg/day
maraviroc	Decrease dose of maraviroc to 150mg 2x/d. Do not use concurrent with itraconazole if ClCr < 30 mL/min.
methylprednisolone	Consider dose reduction and monitor for increased steroid related adverse effects.
midazolam (IV)	consider dose reduction as AUC increases 3fold
mifepristone	When already using itraconazole, start mifepristone at 300mg/d and titrate to 900mg/day if appropriate. When already using mifepristone and itraconazole is initiated, reduce the dose of mifepristone depending on the starting dose from 1200mg to 900mg; 900mg to 600mg; 600mg to 300mg; 300mg to 0mg.
nilotinib	Decrease the nilotinib dose to 300 mg once daily for patients with resistant or intolerant Ph+ CML, or to 200 mg once daily for patients with newly diagnosed Ph+ CML in chronic phase.
olaparib	The dose of olaparib tablets should be reduced to 100 mg twice daily and the dose of olaparib capsules should be reduced to 150 mg twice daily. Patients should be closely monitored for evidence of toxicity. After stopping itraconazole, wait for 3 to 5 times the half-life of itraconazole, and then resume the olaparib dose used prior to use of itraconazole.
palbociclib	Avoid concurrent use; if not possible, reduce dose Palbociclib to 75 mg/day.
panobinostat	Reduce the panobinostat dose to 10 mg when it must be used with itraconazole. Monitor for severe adverse effects related to panobinostat.
pazopanib	Reduce pazopanib dose to 400 mg. Further dose reductions may also be required.

ponatinib	The starting dose of ponatinib should be reduced to 30 mg daily during treatment with itraconazole.
quetiapine	In patients receiving quetiapine, reduce the quetiapine dose to one sixth of the regular dose. Initiate quetiapine at the lowest dose and up-titrate cautiously as needed
ribociclib	Reduce ribociclib dose to 400 mg once daily. With any such concurrent use, monitor closely for evidence of treatment-related toxicity. Following discontinuation of itraconazole in combination with ribociclib, the ribociclib dose should be increased to the dose used prior to use of the inhibitor once 8 days of the discontinued itraconazole has passed. Drugs listed as exceptions to this monograph are discussed in further detail in separate drug interaction monographs.
riociguat	Consider starting with a reduced riociguat (CYP3A4 substraat en p-gp substraat) dose of 0.5 mg three times a day Patients receiving such a combination should also be monitored extra closely for signs or symptoms of hypotension.
ritonavir	Limit the adult maximum itraconazole dose to 200 mg/day in patients receiving ritonavir.
rosuvastatin	Rosuvastatin Canadian product labeling recommends limiting the rosuvastatin dose to a maximum of 20 mg/day in patients receiving itraconazole. Consider increased monitoring for rosuvastatin associated adverse events (e.g., myopathy) in patients receiving concomitant itraconazole and with other predisposing risk factors (e.g., elderly, renal impairment).
ruxolitinib	Reduce ruxolitinib dose by 50% (rounded up to the nearest available tablet strength) if the previous dose was 10 mg twice daily or more, or reduce the dose to 5 mg once daily if the previous dose was 5 mg twice daily. Avoid itraconazole in patients receiving established ruxolitinib doses of 5 mg daily.
saquinavir	Limit the adult maximum itraconazole dose to 200 mg/day in patients receiving saquinavir/ritonavir.

Category C: drugs that require monitoring when administered together with itraconazole

Drug molecule	Comments
afatinib	Monitor closely for signs and symptoms of afatinib toxicity during concomitant treatment with itraconazole. The U.S prescribing information recommends to reduce the afatinib dose by 10 mg if not tolerated and to increase back to the original afatinib dose following discontinuation of P-glycoprotein inhibitors as tolerated. At least some non-US labeling recommends avoiding concurrent use. If such a combination is needed, the P-glycoprotein inhibitor should be given simultaneously with or after the afatinib dose.
aliskiren	Avoid concurrent use; if not possible, monitor for aliskiren toxicity.
amlodipine	Monitor for increased amlodipine effects. Dose reduction may be needed.
amphotericin B	Amphotericin B levels may be decreased. Monitor these levels closely.
bedaquiline	Monitor patients for increased toxic effects of bedaquiline (eg. QTc interval prolongation). Limit concomitant administration of bedaquiline with itraconazole to no more than 14 days, unless the expected benefit of continued administration outweighs the possible risks.
bictegravir	Monitor for increased bictegravir toxicity.
bortezomib	Monitor closely for bortezomib toxicity and reduce dose if necessary.
bosentan	Monitor for decreased effects of itraconazole.
brentuximab vedotin	Monitor for evidence of brentuximab vedotin toxicity.
brinzolamide	Monitor for increased brinzolamide toxicity.
bromperidol	Monitor for changes in therapeutic effects of bromperidol.
budesonide	Avoid concurrent use; if not possible, monitor for corticosteroid toxicity.
buprenorphine	Monitor for increased or prolonged buprenorphine effects. Reduce dose of buprenorphine if necessary.
busulfan	Monitor for increase in serum concentration of busulfan.
cabergoline	Monitor for increased cabergoline effects and toxicities.
calcifediol	Monitor for increased calcifediol effects (eg, hypercalcemia, subtherapeutic intact PTH, elevated 25-hydroxyvitamin D levels). Reduced calcifediol doses may be required.
cannabidiol	Monitor for increased cannabidiol effects/toxicities. Cannabidiol dose reductions may be required.

celiprolol	Monitor for increased celiprolol effects/toxicities (eg, hypotension, bradycardia). Decreased celiprolol doses may be required.
cinacalcet	Monitor intact parathyroid hormone and serum calcium concentrations closely. Cinacalcet dose reductions may be required.
clozapine	Monitor for increased clozapine effects/toxicities.
codeïne	Monitor for respiratory depression and other adverse effects, and consider decreasing the dose of codeine until stable drug effects are achieved. If itraconazole is discontinued, monitor for decreased therapeutic effects of codeine. Monitor for opioid withdrawal and consider increasing the dose of codeine until stable drug effects are achieved. Some non-US labels recommend avoiding this combination when possible.
dabigatran	Monitor closely for evidence of an excessive clinical response to dabigatran (eg, bruising, bleeding). Avoid combination when reduced renal function (CrCL < 30 mL/min for the treatment of atrial fibrillation or if CrCL < 50 mL/min for other dabigatran indications).
Dabrafenib	Avoid concurrent use, if not possible, monitor for reduced clinical response to itraconazole.
deferasirox	Monitor clinical response and serum concentrations when possible, combination could decrease itraconazole serum concentration.
dexamethasone (ophthalmic)	Monitor for signs and symptoms of corticosteroid excess (ie, Cushing syndrome).
diënogest	Monitor patients for signs or symptoms of dienogest adverse effects.
drospirenone	Monitor for hyperkalaemia
dutasteride	Monitor for increased adverse reactions of dutasteride (impotence, decreased libido, breast and ejaculation disorders).
efavirenz	Avoid concurrent use; if not possible, monitor for Efavirenz toxicity.
enzalutamide	The use of itraconazole concurrently with or within 2 weeks of any CYP3A4 inducer is not recommended. If such a combination can not be avoided, monitor patients closely for evidence of diminished clinical response to itraconazole.
enzalutamide	Monitor closely for enzalutamide toxicity.
erlotinib	Monitor the patient closely for the development of severe adverse reactions (e.g., severe diarrhea, severe skin reactions, etc.). If severe adverse reactions do occur, reduce the erlotinib dose (in 50 mg decrements).
etravirine	Monitor for increased effects/toxicity of etravirine if used concomitantly with any azole antifungal agent. Concurrent use of etravirine may necessitate dose adjustment of itraconazole but specific guidelines for dose adjustment are lacking.

fentanyl	Patients receiving fentanyl and itraconazole should be closely monitored for several days following initiation of the combination, and fentanyl dosage reduced as necessary.
fexofenadine	Monitor closely for adverse effects of fexofenadine.
fluticason	Use of orally inhaled fluticasone furoate with strong CYP3A4 inhibitors should be done with caution, and patients using such a combination should be monitored more closely for evidence of systemic corticosteroid toxicities.
galantamine	Monitor for increased effects of galantamine.
gefitinib	Closely monitor for evidence of gefitinib-related adverse effects or toxicity.
haloperidol	Monitor for QTc interval prolongation and ventricular arrhythmias (including torsades de pointes).
ifosfamide	Monitor for reduced ifosfamide therapeutic effects.
imatinib	Monitor for increased side effects of imatinib.
isoniazid	Monitor for decreased effects of itraconazole.
lacosamide	Monitor closely for lacosamide toxicity, especially in case of renal dysfunction or hepatic impairment. Dose adjustments of lacosamide may be necessary.
levobupivacaine	Monitor for levobupivacaine toxicity.
lumefantrine	Monitor for increased toxicity of lumefantrine (including QT prolongation)
medroxyprogester on	Monitor for increased medroxyprogesterone effects/toxicities.
meloxicam	Monitor for reduced effects of meloxicam in patients treated with itraconazole. Meloxicam dose adjustment or use of an alternative anti-inflammatory agent may be necessary.
midostaurin	Avoid concurrent use; if not possible, monitor for increased midostaurin toxicity.
mirtazapine	Monitor for increased mirtazapine effects/toxicities.
mometasone (nasal)	Monitor closely for evidence of signs or symptoms of excessive corticosteroid response (ie. Cushing syndrome).
netupitant	Monitor for increased effects of CYP3A4 substrates (decreased effects for prodrugs activated by CYP3A4) when netupitant is coadministered with a CYP3A4 substrate. Avoid concomitant use of CYP3A4 substrates for 1 week if possible. If not possible, a reduced dose of the CYP3A4 substrate may be necessary. Due to the long half-life of netupitant, the inhibitory effect on CYP3A4 may last for multiple days.

nevirapine	Avoid concurrent use; if not possible, monitor for reduced clinical response to itraconazole.
nintedanib	Monitor patients closely for evidence of nintedanib toxicity when using such a combination. Therapy adjustments may be necessary.
oxybutynin	Monitor for signs of increased oxybutynin effects/toxicity (eg, anticholinergic effects).
oxycodone	Patients should be monitored more frequently for opioid effects (e.g. sedation, respiratory depression) when oxycodone is co-administered with itraconazole. Dosage adjustment of oxycodone may be required.
paliperidone	Closely monitor for increased paliperidone-related toxicity.
parecoxib	Monitor for increased parecoxib effects/toxicities.
phenytoin	Monitor patients closely for azole therapeutic failure/relapse and phenytoin toxicity.
pimecrolimus	Monitor for evidence of pimecrolimus toxicity especially in patients with widespread and/or erythrodermic disease. These patients are likely to experience increased absorption of the pimecrolimus.
piperaquine	Frequent ECG monitoring is recommended due to the increased risk for QTc prolongation associated with increased piperaquine exposure.
pravastatin	Consider enhanced monitoring for pravastatin toxicity (e.g., myalgia, rhabdomyolysis, liver function test abnormalities).
prednisolone	Monitor for increased steroid-related adverse effects.
prednisone	Monitor for increased steroid-related adverse effects.
propafenone	Monitor patients more closely for evidence of propafenone toxicity. Patients using such a combination should avoid the use of any CYP2D6 inhibitor, as the use of propafenone together with both a CYP3A4 inhibitor and a CYP2D6 inhibitor should be avoided due to the potential for an even greater risk for toxicity.
prucalopride	Monitor patients more closely for evidence of toxicity/side effects.
repaglinide	Monitor for increased hypoglycemic response to repaglinide.
rifaximin	Monitor patients for increased incidence of rifaximin (P-gp substrate)-related adverse effects when receiving both rifaximin and a potent P-glycoprotein inhibitor such as itraconazole. In addition, one consequence of this interaction may be an increased likelihood that rifaximin would alter the metabolism of other drugs subject to intestinal and/or hepatic drug metabolism. Patients with underlying hepatic dysfunction and/or gastrointestinal disease may be at a higher risk for a clinically significant interaction.
rilpivirin	Increase monitoring for adverse effects of rilpivirine

rosuvastatin	Consider increased monitoring for rosuvastatin associated adverse events (e.g., myopathy) in patients receiving concomitant itraconazole and with other predisposing risk factors (e.g., elderly, renal impairment). Rosuvastatin Canadian product labeling recommends limiting the rosuvastatin dose to a maximum of 20 mg/day in patients receiving itraconazole.
sarilumab	Monitor for decreased levels/effects of itraconazole in patients initiated on sarilumab.
silodosin	Monitor patients for evidence of increased silodosin (P-gp substrate) adverse effects.
siltuximab	Monitor for decreased levels/effects of itraconazole in patients receiving concurrent siltuximab therapy. Monitor for increased levels/effects of itraconazole with siltuximab discontinuation.
sorafenib	Monitor for signs and symptoms of toxicity of sorafenib
stiripentol	The stiripentol product monograph cautions that concurrent use of stiripentol with CYP3A4 substrates may result in increased concentrations and effects (including an increased risk of adverse effects) of itraconazol, necessitating caution with any concurrent use. Use of stiripentol with CYP3A4 substrates that are considered to have a narrow therapeutic index should be avoided due to the increased risk for adverse effects and toxicity.
sunitinib	Decrease the sunitinib (CYP3A4 subtrate, the product is a primary active metabolite) dose to a minimum of 37.5 mg daily when treating **gastrointestinal stromal tumor (GIST) or renal cell carcinoma (RCC)**. Decrease the sunitinib dose to a minimum of 25 mg daily when treating **pancreatic neuroendocrine tumor (pNET)**. Monitor patients carefully for both evidence of sunitinib toxicity and reduced sunitinib efficacy.
tacrolimus	Monitor clinical tacrolimus (CYP3A4 substrate) response closely and frequently monitor tacrolimus serum concentrations with concurrent use of itraconazole. Tacrolimus dose reductions and/or prolongation of the dosing interval will likely be required. Reduce tacrolimus dose to approximately one-third of the original dose when starting. The magnitude of this interaction may be greater in older patients.
temsirolimus	Decrease temsirolimus (CYP3A4 substrate) dose to 12.5 mg per week and monitor patients for increased temsirolimus effects and toxicities. Once the strong CYP3A4 inhibitor is discontinued, a washout period of approximately 1 week should be allowed before the temsirolimus dose is adjusted back to the dose used prior to initiation of the strong CYP3A4 inhibitor.
tetrahydrocannabinol	Monitor patients who use tetrahydrocannabinol (THC) closely for enhanced effects of THC (e.g., cognitive effects, sedation, dizziness, tachycardia).
thiotepa	Thiotepa (CYP3A4 substrate, prodrug of the active metabolite TEPA): monitor for adverse effects of thiotepa and decreased efficacy of thiotepa

tocilizumab	Monitor for decreased levels/effects of CYP3A4 substrates (e.g., simvastatin) in patients receiving concurrent tocilizumab therapy. Monitor for increased levels/effects of CYP3A4 substrates with tocilizumab discontinuation.
tramadol	Monitor patient closely for evidence of tramadol toxicities
trastuzumab emtansine	Avoid concurrent use; if not possible, monitor for Trastuzumab Emtansin toxicity.
vilanterol	Monitor for increased vilanterol effects and toxicities
vinblastine	Monitor response to vinblastine closely (particularly hematologic and neurologic status) when using vinblastine together with itraconazole
vincristine	Monitor response to vincristine (CYP3A4 substrate) therapy closely when used together with itraconazole. In particular, monitor for signs and symptoms of neurotoxicity, gastrointestinal toxicity, and other vincristine toxicities with this combination. Dose adjustment of vincristine should be considered prior to use with itraconazole, but specific dose adjustment guidelines are not available.
vindesine	Monitor for increased vindesine toxicities
vinorelbine	Monitor for increased vinorelbine effects/toxicities
vit K antagonists	Monitor for increased anticoagulant effects (e.g., INR, bleeding), and decreased effects if itraconazole is discontinued/dose decreased.
zolpidem	Monitor for increased zolpidem effects/toxicities.
zuclopenthixol	Combination of a strong CYP2D6 inhibitor (eg, quinidine, paroxetine, fluoxetine) concomitantly with zuclopenthixol and a strong CYP3A4 inhibitor (i.e. itraconazol) may be at risk for increased zuclopenthixol systemic exposure and toxicity. Zuclopenthixol dose reduction might be considered in these patients to avoid potential toxicity. Whether the same risk applies to individuals with functional CYP2D6 activity receiving zuclopenthixol with a strong CYP3A4 inhibitor isn't clear.

Drug molecule	Comments
alfusozine	
aliskiren	
alprazolam	
aprepitant	
avanafil	
bilastine	Alternatives to bilastine therapy should be considered. Bilastine therapy should be avoided in patients with moderate to severe renal insufficiency receiving itraconazole due to an increased risk of adverse effects of bilastine (e.g. QTc-prolongation).
bosutininb	
bromocriptine	
cobimetinib	
dapoxetine	
disopyramide	
domperidone	
doxorubicin	
eletriptan	
eliglustat	
eplerenone	
ergotamine	
everolimus	
felodipine	
fluticasone	
fosaprepitant	
ibrutinib	Interrupt ibrutinib therapy if short-term treatment with itraconazole is necessary.
idelalisib	
irinotecan	Avoid itraconazole during and within 1 week prior to irinotecan.
isavuconazole	
ivabradine	
lercanidipine	
macitentan	
methadone	
midazolam	
mizolastine	
nimodipine	
pazopanib	
pimozide	
reboxetin	
red yeast rice	Switch red yeast rice to fluvastatin, rosuvastatin, pravastatin.
regorafenib	
rifampicin	
rivaroxaban	

rupatadine	
Saccharomyces boulardii	
salmeterol	
sildenafil	**Pulmonary arterial hypertension (PAH):** combination with itraconazol is not recommended.
silodosin	
simvastatin	Switch simvastatin to fluvastatin, rosuvastatin, pravastatin.
tadalafil	For treatment of **pulmonary arterial hypertension** in patients who are also taking itraconazole
tamsulosine	
temsirolimus	
ticagrelor	
tolvaptan	
topotecan	
trabectedin	If itraconazole is used for less than 14 days, administer itraconazole 1 week after the trabectedin infusion, and discontinue it the day prior to the next trabectedin infusion.
triazolam	
ulipristal	
vemurafenib	Coadministration of itraconazole and vemurafenib increased vemurafenib AUC by approximately 40%
vincristine	
