# Supplementary material for: Itraconazole for COVID-19: preclinical studies and a proof-of-concept randomized clinical trial
Source: eBioMedicine. 2021 Mar 19;66:103288. doi: 10.1016/j.ebiom.2021.103288 (PMC7979145; doi:10.1016/j.ebiom.2021.103288)
Supplement: Supplementary file 3 [file mmc3.docx]

Caption for supplementary material

- Supplementary Table
- Supplementary Figure 1: Additional Preclinical Data
- Supplementary Figure 2: Additional Clinical Data
- Supplementary Methods
- Dawn-Studies List of collaborators
- Study Protocol
- Statistical Analysis Plan
- Arrive Checklist
- Consort Checklist
