# Supplementary material for: Itraconazole for COVID-19: preclinical studies and a proof-of-concept randomized clinical trial
Source: eBioMedicine. 2021 Mar 19;66:103288. doi: 10.1016/j.ebiom.2021.103288 (PMC7979145; doi:10.1016/j.ebiom.2021.103288)
Supplement: Supplementary file 6 [file mmc6.pdf]

DAWN Study

ITRA Specific Statistical Analysis Plan

Final Version 1.0

Project Title: A randomized, open-label, adaptive, proof-of-concept clinical trial of new antiviral drug candidates against SARS-CoV-2 – Direct Antivirals Working against NCoV (DAWN) trial: Evaluation of Itraconazole

Date: 30 June 2020

Version: Final Version 1.0

1 Signature Page

This statistical analysis plan is approved by:

Coordinating Investigator:	30 JUN 2020<br>Date	Prof. Dr. Eric Van Wijngaerden
Statistician:	30 Jun 2020<br>Date	Ann Belmans, M.Sc.
DAWN Coordinator	30-06-2020<br>Date	Prof. Dr. Peter Verhamme

2 Contents

1 Signature Page 2
2 Contents 3
3 Purpose....5
4 Description of the Study 5
5 Master Statistical Analysis Plan....5
6 Study Objectives and Endpoints 5
6.1 Study Objective 5
6.2 Study Endpoints....5
6.2.1 Primary Outcome 5
6.2.2 Secondary Outcomes....6
6.2.3 Long-Term Exploratory Outcomes 6
7 Study Methods 6
7.1 Overall Study Design and Plan....6
7.2 Selection of Study Population 7
7.3 Method of Treatment Assignment and Randomisation 7
7.4 Blinding of Study Treatment 7
8 Sequence of Planned Analyses 7
8.1 Interim Analyses....7
8.2 Final Analysis and Reporting 7
9 Sample Size Determination....7
10 Analysis Populations....7
10.1 Full Analysis Set (FAS)....8
10.2 Safety Set (SS)....8
10.3 Per Protocol Set (PPS)....8
10.4 Analysis Software....8
10.5 Summary Statistics 8
10.6 Statistical Comparisons between Groups 8
10.7 Selective Randomisation 9
10.8 Period Effect....9
10.9 Centre Effect....9
10.10 Choice of Controls....9
10.11 Factorial Design....9
10.12 Methods for Withdrawals, Missing Data and Outliers 9
10.13 Data Transformations 10
10.14 Multicentre Study 10

10.15 Stratification Factors 10
10.16 Multiple Comparisons 10
10.17 Planned Subgroups, Interactions and Covariates 10
11 Study Subjects 11
11.1 Disposition of Subjects and Withdrawals 11
11.2 Protocol Violations and Deviations 11
12 Demographics and Other Baseline Characteristics 11
13 Primary Endpoints 12
13.1 Primary Efficacy Endpoints 12
13.1.1 Cumulative Clinical Status from Day 1-15 12
14 Secondary Efficacy Endpoints 12
14.1 Clinical Status During Hospitalisation, on Day 15 and 29 12
14.2 All-Cause Mortality on Day 15 and Day 29 12
14.3 Time to Sustained Clinical Improvement or Discharge 13
14.4 Incidence and Duration of Supplemental Oxygen 13
14.5 Incidence and Duration of Mechanical Ventilation 13
14.6 Duration of Hospital Stay 13
14.7 Incidence and Duration of ICU Stay 14
14.8 Daily NEWS score 14
14.9 Combined Cardiac Endpoint 14
14.10 QTc data 14
14.11 Laboratory Data, and other Markers 14
15 Exploratory Outcomes 15
15.1 PCR for SARS-CoV-2 15
15.2 Itraconazale Trough Levels on Day 3 and 6 15
16 Adverse Events 15
17 Other Data 15
18 PK/PD Analyses 15
19 References 15
20 List of Tables and Figures 16
Appendix I: PK/PD Statistical Analysis Plan 19

3 Purpose

In addition to the Global SAP, this document provides further details for the statistical evaluation of the primary, secondary and exploratory endpoints of the ITRA compound within the DAWN study

4 Description of the Study

A total of 200 patients were to be randomised in a 1:1 ratio to either Standard of Care (SOC) or Itraconazole.

When 68 patients were randomised, the study was put on hold by the DSMB due to what they believed were low plasma levels for the study drug.

Following additional results from in-vitro and pre-clinical studies, the decision was taken not to restart the study.

5 Master Statistical Analysis Plan

Due to the fast-changing addition of novel treatments and hypotheses of interest, a Master SAP describes the general statistical methods to be used for statistical evaluation of the primary, secondary, exploratory and safety endpoints of all compounds within the DAWN study.

This ITRA-Specific SAP provides additional and specific details about the statistical methods for the evaluation of the outcomes for Itraconazole at the time of early termination.

6 Study Objectives and Endpoints

6.1 Study Objective

The objective of the DAWN study is to evaluate the efficacy of various compounds of interest in the treatment of hospital-admitted COVID-19 patients.

6.2 Study Endpoints

6.2.1 Primary Outcome

The primary outcome for the evaluation of Itraconazole is:

<u>Cumulative Clinical Status of Day 1 to 15</u>

Daily assessment of Clinical Status up to Day 15 on a 7-point ordinal scale

1. Not hospitalized, no limitations on activities;
2. Not hospitalized, limitations on activities;
3. Hospitalized, not requiring supplemental oxygen;
4. Hospitalized, requiring supplement oxygen;
5. Hospitalized, on non-invasive ventilation or high flow oxygen devices;
6. Hospitalized, on invasive mechanical ventilation or ECMO;
7. Death.

6.2.2 Secondary Outcomes

The following secondary outcomes will be of interest:

1. Daily clinical status during hospitalization and on Days 15 and 29;
2. All-cause mortality on Days 15 and 29;
3. Time to clinical improvement (first 2-point improvement from highest previously recorded in clinical status);
4. Incidence and duration of supplemental oxygen;
5. Incidence and duration of mechanical ventilation;
6. Duration of hospital stay;
7. Duration of ICU stay;
8. Time to Death;
9. NEWS assessed daily during hospitalization and on Days 15 and 29;
10. Adverse events graded as severe or SAEs
11. Laboratory data assessed on days 1, 3, 5, 8, 11, 15 and 29.
12. Combined cardiac endpoint: any of the following: hsTrop>0.5 ng/mL, ventricular arrhythmia requiring intervention, reanimation, sudden cardiac death.
13. QTc and changes in QTc at baseline and follow-up at day 2-3.

In addition, although not prescribed in the protocol, the primary endpoint for the other DAWN compounds will be analysed as a secondary outcome:

14. Time to sustained clinical improvement or life discharge: time from Day 0 to sustained clinical improvement or life discharge, whichever comes first, whereby a sustained clinical improvement is defined as an improvement of $\geq$ 2 points vs the highest value of Day 0 and 1 and sustained for at least 3 days.

6.2.3 Long-Term Exploratory Outcomes

1. Qualitative and quantitative PCR for SARS-CoV-2 in swab on Day 1 and 6.
2. Itraconazole trough level on Day 6

7 Study Methods

7.1 Overall Study Design and Plan

The DAWN study evaluates several potential candidates in a randomised design vs Standard of Care (SOC) as a control group.

In the ITRA sub-protocol, patients are randomised in a 1:1 ratio to either Itraconazole or Standard of Care (SOC).

7.2 Selection of Study Population

Adult patients who tested positive for SARS-CoV-2 and are admitted to the hospital are screened for eligibility. Once eligibility is confirmed, informed consent is sought from the patient to participate in the study. Patients who consented are randomised to either Itraconazole or SOC.

7.3 Method of Treatment Assignment and Randomisation

Patients are randomised in a 1:1 ratio, stratified on baseline severity (severe/non-severe) of disease.

7.4 Blinding of Study Treatment

This was an open-label study, meaning both patients and study personnel were aware of the assigned treatment.

8 Sequence of Planned Analyses

8.1 Interim Analyses

No formal interim to check for early efficacy or futility were foreseen.

A regular review of the safety data was performed by a Data Safety Monitoring Board.

8.2 Final Analysis and Reporting

Upon final database lock, statistical analyses of the data will be performed according to the methods described in this document and the Global SAP.

Any deviations will be documented. The analysis populations and analysis plan will be finalised at a Blinded Review Meeting prior to database lock where all attendees will be blind to the randomised study treatment.

9 Sample Size Determination

Based on the clinical scores on day 7 and day 15 from recently published data[14], we estimate a mean cumulative clinical severity score up to day 15 of 60, with a standard deviation of 20. With a power of 0.8 and an alpha of 0.05, sample size estimates to detect an 8-point difference in cumulative clinical severity score would require 198 patients with a 1:1 randomisation (2 times 99).

A total of 200 patients were to be randomized.

However, the study was put on hold by the DSMB when 68 patients had been randomised. Based on additional results from in-vitro and pre-clinical studies, the study was not restarted.

10 Analysis Populations

The following analysis sets will be of interest:

10.1 Full Analysis Set (FAS)

The FAS will include all randomised patients according to their randomised treatment who did not violate the following eligibility criteria:

a) Covid-negative patients: inclusion criterion 4
b) Taking medication with known interaction with study drug: relevant part of exclusion criterion 5.

The FAS will be used for the evaluation of all efficacy endpoints.

10.2 Safety Set (SS)

The SS will include all patients from FAS according to their actual treatment. Patients randomised to the interventional group who did not receive any study treatment will be included in the SOC group.

The SS will be used for the evaluation of all safety parameters.

10.3 Per Protocol Set (PPS)

No Per Protocol Set will be defined.

10.4 Analysis Software

All analyses will be performed using SAS software version 9.41 or higher for Windows 10 or higher.

10.5 Summary Statistics

Continuous variables will be summarized by treatment group by the number of non-missing data points, mean, standard deviation, median and interquartile range.

Categorical and ordinal variables will be summarized by treatment group by observed frequencies and percentages relative to the total number of non-missing items.

All summary statistics will be presented by treatment group and, where possible, overall.

Data collected at several time points during the trial will be presented by planned visit, regardless of when the visit actually took place.

If applicable, changes from ‘baseline’ will be calculated whereby ‘baseline’ is defined as the last available measurement prior to randomisation, unless specified otherwise.

Day 0 is defined as the day of randomization.

10.6 Statistical Comparisons between Groups

Unless specified otherwise, the following methods will be used to compare treatment groups:

- <u>Normally distributed continuous data</u>: 2-sample t-test
- <u>Continuous data showing serious deviations from normal distribution</u>: Wilcoxon rank-sum test.

- Categorical data: chi-square or Fisher's exact test if cells with expected counts of <5 patients.
- Ordinal data: Wilcoxon rank-sum test or chi-square test for trend.
- Survival data: log-rank test
- Competing risk data: Gray's test

For each treatment comparison of interest, the treatment effect will be estimated by an appropriate measure (i.e., difference of the means, odds ratio, risk ratio, hazard ratio, …) and presented along with its associated 95% confidence interval.

To take account for the stratification, all statistical comparisons of efficacy endpoints will be adjusted for baseline disease severity.

10.7 Selective Randomisation

Not applicable.

10.8 Period Effect

Not applicable.

10.9 Centre Effect

This was a single-centre study.

10.10 Choice of Controls

Not applicable.

10.11 Factorial Design

Not applicable.

10.12 Methods for Withdrawals, Missing Data and Outliers

Clinical Status will be recorded for all patients up to Day 15, regardless of whether or not they were discharged from the hospital prior to Day 15 or not. For patients still hospitalized at Day 15, Clinical Status will be recorded until the end of their hospital stay. At Day 29, all patients are contacted in order to obtain their Clinical Status on that day.

Patients who do not have complete **Clinical Status** data up to Day 29 will be accounted for using the following steps:

I. Stage 1: **Single day missing, with preceding and following day known**: in these cases, the missing value will be imputed by the maximum of the two surrounding values.
II. Stage 2: **Patients with data recorded on Day 29 regarding Clinical Status and Rehospitalisations**: in the available observed data, all overall transitional probabilities between status 1 and 2 will be calculated (e.g. when a patient has status 1 on a certain day: what is probability of remaining in 1 or transitioning to 2 the next day). These resulting probabilities will be used to multiply impute missing out-of-hospital data prior to Day 29,

based on the data observed or imputed on the previous day. A total of 100 imputations will be performed and 1323 will be used as seed number.

III. Stage 3: The remaining missing data will be imputed using multiple imputation methodology. The fully conditional specification method will be used with a multinomial logistic regression and will be done in a consecutive manner as follows:

1. Step 1: impute Day 1 based on clinical variables and Day 0;
2. Step 2: impute Day 2 based on clinical variables and imputed Day 1 status
3. Step 3: impute Day 3 based on clinical variables and imputed status at Days 1-2;
4. Etc…

This method will be used up to day 29, whereby again 1323 will be used as seed number in each step.

The clinical variables that will be included in the imputation model will be the following:

- Randomised treatment group
- Baseline disease severity (stratification factor; severe vs non-severe)
- Oxygen flow of the previous day (only up to day 15)
- CRP recorded on the previous day (only up to day 15

10.13 Data Transformations

When necessary, a log-transformation can be applied to the data in order to satisfy the normality assumption when analyzing data using a general linear model.

10.14 Multicentre Study

Not applicable.

10.15 Stratification Factors

Randomisation was stratified for baseline disease severity. Therefore, all models that are used for the estimation of treatment effects will be adjusted for disease severity.

10.16 Multiple Comparisons

Since only 1 primary endpoint is defined, no adjustment of the significance level is required.

For secondary efficacy endpoints, due to the exploratory nature of the efficacy analyses, no adjustment for multiple comparisons will be made.

10.17 Planned Subgroups, Interactions and Covariates

Subgroup analyses will be performed for the following outcomes:

- Cumulative Clinical Status up to Day 15
- Sustained Clinical Improvement or Discharge.

The following subgroups will be of interest:

1. duration of symptoms prior to enrolment,

2. age groups,
3. disease severity at baseline

Appropriate summary statistics per treatment and estimated treatment differences, will be presented for each subgroup. In addition, the interaction between the above subgroups and randomized treatment will be tested to assess whether the treatment effect differs according to subgroup. In addition, the interaction between randomised treatment and the subgroup will be evaluated. The treatment difference per subgroup will be estimated from an appropriate statistical model (e.g., ANOVA, ANCOVA, logistic regression, Cox regression, …) that includes a factor for treatment, subgroup and their interaction.

Subgroup analyses will only be defined for the FAS.

11 Study Subjects

11.1 Disposition of Subjects and Withdrawals

A summary by treatment group will be provided for the following:

- Number of randomized subjects,
- Number in Full Analysis Set (FAS);
- Number of treated subjects
- Number of subjects included in Safety Set (SS)
- Number of subjects who died in hospital up to Day 15
- Number of subjects who were discharged up to Day 15
- Number of subjects who died out-of-hospital up to Day 15
- Number of subjects who died in hospital up to Day 29
- Number of subjects who were discharged up to Day 29
- Number of subjects who died out-of-hospital up to Day 29

11.2 Protocol Violations and Deviations

Not applicable.

12 Demographics and Other Baseline Characteristics

All data recorded at baseline will be summarized by treatment group and compared using the methods described in Section 7.3.

Summaries will be presented for FAS, SS and PPS separately.

The following baseline information will be presented:

- Demographic characteristics
- Medical history
- Prior medications
- Hospital admission: symptoms
- Imaging data at hospital admission
- Vital signs at hospital admission
- Laboratory data at hospital admission

- ECG at hospital admission

13 Primary Endpoints

13.1 Primary Efficacy Endpoints

The analysis of the primary outcome will be done on the FAS and, if defined, the PPS.

13.1.1 Cumulative Clinical Status from Day 1-15

Cumulative Clinical Status will be calculated from the imputed Clinical Status data.

Cumulative Clinical Status up to Day 15 will be analysed by means of a general linear model. The treatment effect will be estimated by the difference between groups and presented together with its 95% confidence interval.

The general linear model will include a factor for randomised treatment, disease severity and clinical status at baseline (Day 0).

14 Secondary Efficacy Endpoints

Summaries and statistical analyses of the secondary endpoints will be done using the Full Analysis Set.

14.1 Clinical Status During Hospitalisation, on Day 15 and 29

Daily Clinical Status (imputed) will be analysed by means of a proportional odds logistic regression model, performed on each day. The treatment effect will be estimated by the common odds ratio.

The proportional odds logistic regression model will include a factor for randomised treatment and disease severity.

14.2 All-Cause Mortality on Day 15 and Day 29

Mortality rates over time will be estimated by Kaplan-Meier methodology. Confidence intervals will be calculated using the log-log transformation of the standard error. Comparisons of the curves will be done using a log-rank test.

Median times (or other more suitable quantiles) will be presented by treatment group.

The treatment effect will be estimated as a hazard ratio, obtained using a Cox proportional hazards regression model.

The Cox regression will include a factor for randomised treatment and disease severity.

For the analysis of mortality on Day 15, all data beyond Day 15 will be censored at Day 15. Likewise, for the analysis of all-cause mortality on Day 29, all data beyond Day 29 will be censored.

14.3 Time to Sustained Clinical Improvement or Discharge

Time to Sustained Clinical Improvement or Discharge will be derived from the imputed Clinical Status data.

Time to clinical improvement will be analysed using methods for the analysis of competing risk data: the event of interest is clinical improvement, death without improvement will be considered to be the competing risk, patients for whom follow-up ended without clinical improvement will be censored. Comparisons of the CIF curves will be done using Gray's test.

Event rates over time will be estimated as cumulative incidence functions (CIF) and presented along with their 95% confidence intervals.

Median times (or other more suitable quantiles) will be presented by treatment group.

The treatment effect will be estimated by the subdistribution hazard ratio obtained from a Fine&Gray model.

The Fine&Gray regression will include a factor for randomised treatment and disease severity.

14.4 Incidence and Duration of Supplemental Oxygen

Incidence and duration of supplemental oxygen will be calculated from the imputed Clinical Status data.

Incidence of supplemental oxygen will be analysed using competing risk methodology as described in Section 13.2.2 above, whereby death without the administration of supplement oxygen is considered a competing risk.

Competing risk methodology will also be used to evaluate the duration of the supplemental oxygen. The event of interest will be the end of supplemental oxygen when alive, competing risk is the end of supplemental oxygen because of death.

Two separate analyses will be done for the duration: first, patients who did not have any supplemental oxygen will be excluded from the analysis. Second, patients who did not have any supplemental oxygen will be included with a duration of zero days.

14.5 Incidence and Duration of Mechanical Ventilation

Incidence and duration of mechanical ventilation will be calculated from the imputed Clinical Status data.

The statistical methodology for the evaluation of this endpoint will be the same as for incidence and duration of supplemental oxygen (see Section 14.2.4).

14.6 Duration of Hospital Stay

Duration of hospital stay with be analysed by means of competing risk methodology as described in Section 14.2.4 with in-hospital death the competing risk.

14.7 Incidence and Duration of ICU Stay

Incidence of ICU stay will be analysed using the competing risk methodology described in Section 14.2.4, considering death

Duration of ICU stay with be analysed by means of competing risk methodology as described in Section 14.2.4 with in-ICU death the competing risk. For patients who have multiple ICU stays, the durations will be added up.

14.8 Daily NEWS score

Daily NEWS scores will be summarised by treatment group and study day.

14.9 Combined Cardiac Endpoint

The combined cardiac endpoint will be analysed using a logistic regression from which the treatment effect will be estimated as an odds ratio.

The logistic regression will include a factor for randomised treatment and baseline disease severity.

14.10 QTc data

The above parameters will be analysed using a general estimating equation (GEE) model that models all data over time, using an identity link-function and normally distributed errors. A GEE analysis is quite robust against deviations from the distributional assumptions. However, when serious deviations are observed, a log-transformation of the D-Dimer data can be considered.

The GEE model will include all measurements and will include factors for time, treatment, their interaction, baseline disease severity and the baseline value as a covariate. An appropriate variance-covariance matrix will be used to account for interdependencies between the timepoints.

From the GEE model, treatment differences will be calculated at all timepoints and presented along with their 95% confidence interval.

In addition, an overall ‘average’ treatment effect will be estimated by removing the interaction from the model.

The analysis of the QTc data will be done on the Safety Set rather then the Full Analysis Set.

14.11 Laboratory Data, and other Markers

Laboratory data and other markers will be summarised by visit and treatment using appropriate summary statistics.

In addition, statistical comparisons will be performed for the following parameters:

1. CRP
2. D-Dimer
3. Ferritine

The above parameters will be analysed using the methodology described in Section 14.10.

15 Exploratory Outcomes

15.1 PCR for SARS-CoV-2

Viral load will be analysed using using the methodology described in Section 14.10.

In addition, the number (%) of patients that were positive for SARS-CoV-2 will be analysed using logistic regression analyses, including baseline results, treatment group and disease severity as factors in the model.

15.2 Itraconazale Trough Levels on Day 3 and 6

Itraconazole levels will be summarised by treatment group and study day.

16 Adverse Events

The number of events and the number of patients experiencing adverse events will be summarized by treatment group

Summaries of adverse events will be presented for the SS.

17 Other Data

All other data will be summarized treatment group and, if applicable, by day.

18 PK/PD Analyses

A separate PK/PD report will be produced outside the main statistical report. All planned PK/PD analyses will be described in the PK/PD Statistical Analysis Plan (see Appendix I).

19 References

1. SAS software, version 9.4 of the SAS System for Windows. Copyright © 2002 SAS Institute Inc. SAS and all other SAS Institute Inc. product or service names are registered trademarks or trademarks of SAS Institute Inc., Cary, NC, USA
2. Liang KY, Zeger S Longitudinal data analysis of continuous and discrete responses for pre–post designs. Sankhya: The Indian Journal of Statistics (Series B) 2000; 62:134–148.

20 List of Tables and Figures

Note: This list of tables and figures serves as a recommended list to be used across all trials. However, modifications are permissible as endpoints or CRFs differ in individual trials.

Table	1	Disposition & Baseline Information
Table	1.1	Screening Failures
Table	1.2	Subject Disposition
Table	1.3	Demographics
Table	1.3.1	Demographics (FAS)
Table	1.3.2	Demographics (SS)
Table	1.4	Medical History
Table	1.4.1	Medical History (FAS)
Table	1.4.2	Medical History (SS)
Table	1.5	Baseline Medication
Table	1.5.1	Baseline Medication (FAS)
Table	1.5.2	Baseline Medication (SS)
Table	1.6	Admission Signs and Symptoms
Table	1.6.1	Admission Signs and Symptoms (FAS)
Table	1.6.2	Admission Signs and Symptoms (SS)
Table	1.7	Imaging At Admission
Table	1.7.1	Imaging At Admission (FAS)
Table	1.7.2	Imaging At Admission (SS)
Table	1.8	Vital Signs At Admission
Table	1.8.1	Vital Signs At Admission (FAS)
Table	1.8.2	Vital Signs At Admission (SS)
Table	1.9	Laboratory Values At Admission
Table	1.9.1	Laboratory Values At Admission (FAS)
Table	1.9.2	Laboratory Values At Admission (SS)
Table	1.10	ECG At Admission
Table	1.10.1	ECG At Admission (FAS)
Table	1.10.2	ECG At Admission (SS)
Table	1.11	Disease Triage At Admission and Time Differences
Table	1.11.1	Disease Triage At Admission and Time Differences (FAS)
Table	1.11.2	Disease Triage At Admission and Time Differences (SS)
Table	1.12	Respiratory Support During First Two Hours After Admission
Table	1.12.1	Respiratory Support During First Two Hours After Admission (FAS)
Table	1.12.2	Respiratory Support During First Two Hours After Admission (SS)
Table	1.13	Medication Started At Admission
Table	1.13.1	Medication Started At Admission (FAS)
Table	1.13.2	Medication Started At Admission (SS)
Table	2	Hospital Stay
Table	2.1	Duration of Hospital and ICU Stay
Table	2.1.1	Duration of Hospital and ICU Stay (FAS)
Table	2.1.2	Duration of Hospital and ICU Stay (SS)
Table	2.2	Bar Chart of Hospital Status per Day
Figure	2.2.1	Bar Chart of Hospital Status per Day (FAS)
Figure	2.2.2	Bar Chart of Hospital Status per Day (SS)
Table	3	Study Drug
Table	3.1	Study Drug Administration
Table	3.1.1	Study Drug Administration (FAS)
Table	3.1.2	Study Drug Administration (SS)
Table	3.2	Plasma Concentrations

Table	3.2.1	Plasma Concentrations (FAS)
Table	3.2.2	Plasma Concentrations (SS)
Table	4	Other In-Hospital Treatment Data
Table	4.1	Respiratory Support During Hospital Stay
Table	4.1.1	Respiratory Support During Hospital Stay (FAS)
Table	4.1.2	Respiratory Support During Hospital Stay (SS)
Table	4.2	Medications During Hospital Stay
Table	4.2.1	Medications During Hospital Stay (FAS)
Table	4.2.2	Medications During Hospital Stay (SS)
Table	5	Primary Endpoint
Table	5.1	Cumulative Clinical Score up to Day 15 (FAS)
Table	6	Secondary Endpoint: Daily Clinical Status
Table	6.1	Clinical Status by Day (FAS)
Figure	6.2	Bar Chart of Daily Clinical Status (FAS)
Table	7	Secondary Endpoint: Sustained Clinical Improvement or Discharge
Table	7.1	Estimated Event Rates and Hazard ratio (FAS)
Figure	7.2	Cumulative Incidence Curves
Table	8	Secondary Endpoint: All-Cause Mortality on Day 15 and Day 29
Table	8.1	Estimated Event Rates and Hazard Ratios
Figure	8.2	Kaplan-Meier Curve for All-Cause Mortality up to Day 15
Figure	8.3	Kaplan-Meier Curve for All-Cause Mortality up to Day 29
Table	9	Secondary Endpoint: Incidence and Duration of Supplemental Oxygen
Table	9.1	Incidence: Estimated Event Rates and Hazard ratio
Figure	9.2	Incidence: Cumulative Incidence Curves
Table	9.3	Duration for All Patients: Estimated Event Rates and Hazard ratio
Figure	9.4	Duration for All Patients: Cumulative Incidence Curves
Table	9.5	Duration for Patients with SO2: Estimated Event Rates and Hazard ratio
Figure	9.6	Duration for Patients with SO2: Cumulative Incidence Curves
Table	10	Secondary Endpoint: Incidence and Duration of Mechanical Ventilation
Table	10.1	Incidence: Estimated Event Rates and Hazard ratio
Figure	10.2	Incidence: Cumulative Incidence Curves
Table	10.3	Duration for All Patients: Estimated Event Rates and Hazard ratio
Figure	10.4	Duration for All Patients: Cumulative Incidence Curves
Table	10.5	Duration for Patients with MV: Estimated Event Rates and Hazard ratio
Figure	10.6	Duration for Patients with MV: Cumulative Incidence Curves
Table	11	Secondary Endpoint: Duration of Hospital Stay
Table	11.1	Estimated Event Rates and Hazard ratio
Figure	11.2	Cumulative Incidence Curves
Table	12	Secondary Endpoint: Incidence and Duration of ICU Stay
Table	12.1	Incidence: Estimated Event Rates and Hazard ratio
Figure	12.2	Incidence: Cumulative Incidence Curves
Table	12.3	Duration for All Patients: Estimated Event Rates and Hazard ratio
Figure	12.4	Duration for All Patients: Cumulative Incidence Curves
Table	12.5	Duration for ICU Patients: Estimated Event Rates and Hazard ratio
Figure	12.6	Duration for ICU Patients: Cumulative Incidence Curves
Table	13	Secondary Endpoint: Daily NEWS Scores
Table	13.1	Daily Scores
Figure	13.2	Box Plots of Scores by Study Day
Table	14	Secondary Endpoint: Combined Cardiac Endpoint
Table	14.1	Number (%) of Patients and Estimated Odds Ratio
Table	15	Secondary Endpoint: QTc Value
Table	15.1	QTc Values and Treatment Differences
Figure	15.2	Box Plots of QTc Values by Study Day
Table	15	Secondary Endpoint: Laboratory Data
Table	15.1	Laboratory Data and Treatment Differences
Figure	15.2	Box Plots of QTc Values by Study Day

Table	16	Exploratory Outcome: PCR for SARS-CoV-2: Viral Load
Table	16.1	Viral Load and Treatment Differences
Figure	16.2	Box Plot of Viral Load
Table	16.3	Number (%) of Patients Positive for SARS-CoV-2
Figure	16.4	Proportion of Patients Positive for SARS-CoV-2
Table	17	Exploratory Outcome: Itraconazole Trough Levels
Table	17.1	Summary of Trough Levels
Figure	17.2	Box Plot of Trough Levels
Table	18	Disease-Specific Data
Table	18.1	Symptoms During Hospital Stay
Table	18.2	SIGNS
Table	19	Safety Outcomes
Table	19.1	Laboratory Data (SS)
Table	19.3	Combined Cardiac Endpoint (SS)
Table	19.4	Adverse Events
Table	19.4.1	Number of Patients with Adverse Events (SS)
Table	19.4.2	Number of Patients with Serious Adverse Events (SS)
Table	19.4.3	Number of Patients with Treatment-Related Adverse Events (SS)
Table	19.4.4	Number of Patients with Treatment-Related Serious Adverse Events (SS)
Table	19.5	Other Safety Data
Table	19.5.1	ECG Data (SS)
		Imaging Data (SS)
Table	20	Concomitant Medications
Table	21	Other Data
Table	21.1	Pathogen Testing (FAS)

Appendix I: PK/PD Statistical Analysis Plan
